# A_2A_ Adenosine Receptor Antagonists: Are Triazolotriazine and Purine Scaffolds Interchangeable?

**DOI:** 10.3390/molecules27082386

**Published:** 2022-04-07

**Authors:** Andrea Spinaci, Catia Lambertucci, Michela Buccioni, Diego Dal Ben, Claudia Graiff, Maria Cristina Barbalace, Silvana Hrelia, Cristina Angeloni, Seyed Khosrow Tayebati, Massimo Ubaldi, Alessio Masi, Karl-Norbert Klotz, Rosaria Volpini, Gabriella Marucci

**Affiliations:** 1 Medicinal Chemistry Unit, School of Pharmacy, University of Camerino, Via Madonna delle Carceri, 62032 Camerino, Italy; andrea.spinaci@unicam.it (A.S.); catia.lambertucci@unicam.it (C.L.); michela.buccioni@unicam.it (M.B.); diego.dalben@unicam.it (D.D.B.); gabriella.marucci@unicam.it (G.M.); 2 Department of Chemistry, Life Sciences and Environmental Sustainability, University of Parma, Parco Area delle Scienze, 17/A, 43124 Parma, Italy; claudia.graiff@unipr.it; 3 Department for Life Quality Studies, Alma Mater Studiorum, University of Bologna, Corso d’Augusto 237, 47921 Rimini, Italy; maria.barbalace2@unibo.it (M.C.B.); silvana.hrelia@unibo.it (S.H.); cristina.angeloni@unibo.it (C.A.); 4 Pharmacology Unit, School of Pharmacy, University of Camerino, Via Madonna delle Carceri, 62032 Camerino, Italy; khosrow.tayebati@unicam.it (S.K.T.); massimo.ubaldi@unicam.it (M.U.); 5 Departiment of Neuroscience, Psychology, Drug Research and Child’s Health, NEUROFARBA, Università di Firenze, 50139 Firenze, Italy; alessio.masi@unifi.it; 6 Institut für Pharmakologie und Toxikologie, University of Würzburg, Versbacher Straße 9, D-97078 Würzburg, Germany; klotz@toxi.uni-wuerzburg.de

**Keywords:** A_2A_ adenosine receptor antagonists, purine derivatives, triazolotriazine derivatives, anti-Parkinson agents, anti-inflammatory agents, molecular modeling

## Abstract

The A_2A_ adenosine receptor (A_2A_AR) is one of the four subtypes activated by nucleoside adenosine, and the molecules able to selectively counteract its action are attractive tools for neurodegenerative disorders. In order to find novel A_2A_AR ligands, two series of compounds based on purine and triazolotriazine scaffolds were synthesized and tested at ARs. Compound **13** was also tested in an in vitro model of neuroinflammation. Some compounds were found to possess high affinity for A_2A_AR, and it was observed that compound **13** exerted anti-inflammatory properties in microglial cells. Molecular modeling studies results were in good agreement with the binding affinity data and underlined that triazolotriazine and purine scaffolds are interchangeable only when 5- and 2-positions of the triazolotriazine moiety (corresponding to the purine 2- and 8-positions) are substituted.

## 1. Introduction

The A_2A_ adenosine receptor (A_2A_AR) subtype is one of the four G protein-coupled receptors (GPCRs,) named A_1_, A_2A_, A_2B_, and A_3_, which are ARs activated by the natural nucleoside adenosine (Ado) [1,2]. In contrast to the A_1_ and A_3_ receptor subtypes, which are coupled to G inhibitory proteins, the A_2A_AR and A_2B_AR are coupled to G stimulatory proteins and their activation produces an increase in the second messenger cAMP [3]. It is well-known that Ado is among the key modulators in the manifestation of the periphery and central nervous system (CNS) inflammation. This activity of Ado is mainly due to the activation of the A_2A_AR among the AR subtypes. In the periphery, Ado reduces inflammation through the decreased recruitment of leucocyte [4,5,6]. Regarding the CNS, several studies have demonstrated that A_2A_AR antagonists play a key role in counteracting neuroinflammation [7,8,9], a common event in neurodegenerative diseases, such as PD, characterized by the activation of microglial cells associated with the release of pro-inflammatory mediators [10,11]. In this context, molecules able to selectively block the A_2A_ARs are attractive tools useful in the treatment of neurodegenerative disorders, such as Parkinson’s disease (PD) [12,13]. In fact, numerous studies demonstrated that A_2A_AR antagonists increase the therapeutic efficacy of levodopa (L-Dopa), which represents the election drug for PD therapy [14].

**Figure 1:**
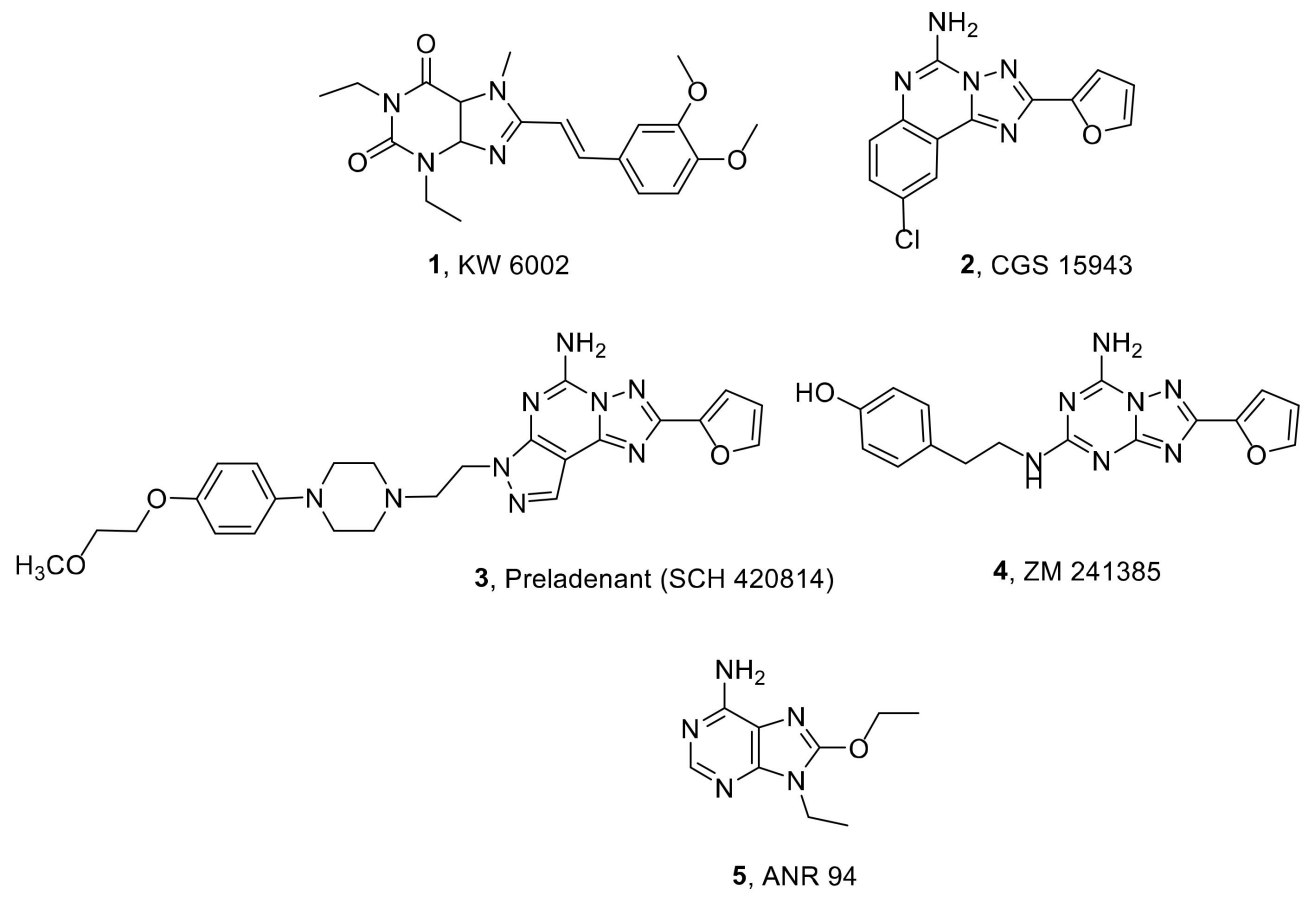
Structures of A_2A_AR antagonists.

These findings led to the commercialization of the first and unique A_2A_AR antagonist Istradefylline (**1**, also called KW6002, Figure 1) as a coadjutant of L-Dopa for the treatment of PD [15]. In 2013, in Japan, Istradefylline was approved under the name Nouriast^®^ [16], and in 2019, the Food and Drug Administration (FDA) approved its commercialization (Nourianz^®^) [17,18]. This molecule belongs to the class of xanthine derivatives such as caffeine and theophylline, which represent the first A_2A_AR antagonists to be discovered [19,20,21]. In recent decades, a great number of AR antagonists were synthesized using different scaffolds. CGS 15943 (**2**, Figure 1) is an example of a triazoloquinazoline derivative, initially proposed as a selective A_2A_AR antagonist, which demonstrated only slight selectivity in subsequent studies [22]. Modification of the CGS 15943 triazoloquinazoline scaffold, obtained by the replacement of the phenyl ring with a pyrazole moiety, considerably improved the selectivity for A_2A_AR, leading to a number of potent and selective A_2A_AR antagonists such as Preladenant (**3**, also called SCH 420814, Figure 1) [23]. This compound has been studied for the treatment of Parkinson’s disease by Schering-Plough (https://www.fiercebiotech.com/biotech/schering-plough-reports-preladenant-meets-primary-endpoint-phase-ii-dose-finding-trial-for, accessed on 8 March 2022) and, later on, it was studied by the Merck Company in two phase III trials as an adjunct to levodopa in PD patients. However, in these studies, its efficacy was not significantly higher than the placebo [24,25]. 

A simplification of the triazolochinazoline scaffold led to the triazolotriazine ZM241385, which binds the A_2A_AR with high affinity (**4**, Ki = 1.6 nM, Figure 1) and shows good selectivity versus the A_1_ and A_3_ ARs but not versus the A_2B_AR. This compound was used as a radioligand for the study of the A_2A_AR both in tritiated and iodinated form and as a ligand for crystallization studies of the A_2A_AR [26,27,28].

Examples of other non-xanthine A_2A_AR antagonists are adenine derivatives, which have been the subject of our research for a long time. In fact, we synthesized a number of such molecules substituted at the 2-, 9-, and/or 8-positions, which were found to behave as A_2A_AR antagonists endowed with various degrees of affinity and selectivity for ARs [29]. Among them, the 9-ethyl-8-ethoxyadenine (ANR 94, **5**, Figure 1) showed good affinity and selectivity for the human A_2A_AR subtype (Ki A_1_ = 2400 nM, Ki A_2A_ = 46 nM, Ki A_2B_ > 30,000 nM, and Ki A_3_ = 21,000 nM) and demonstrated very high efficacy in in vivo models of Parkinson’s disease. In fact, it reversed the catalepsy induced by haloperidol and induced contralateral turning behavior in rats sensitized to L-DOPA. Moreover, this compound showed good efficacy in lowering the intensity of the tremor evoked by Parkinson’s induced by tacrine and in reducing the motor deficit in a neuronal injury model induced by 6-hydroxydopamine [30]. Despite these promising results, ANR 94 does not show a remarkable affinity for A_2A_ARs (Ki = 46 nM) with respect to other A_2A_AR antagonists like as ZM241385, which is considered the reference compound for studying the A_2A_AR.

**Figure 2:**
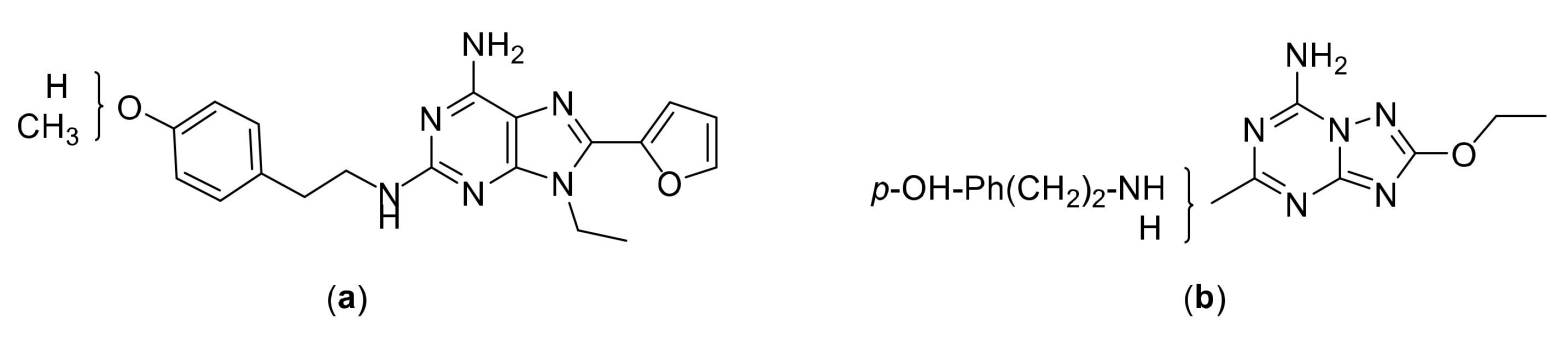
Structures of the designed compounds: (**a**) purine derivatives; (**b**) triazolotriazine derivatives.

Starting from these observations, and in order to verify whether the triazolotriazine scaffold of the ZM241385 can be replaced by the purine ring to give a high affinity A_2A_AR ligand, we synthesized an adenine derivative bearing the same substituents present in the 2- and 5-positions of the ZM241385 structure. Hence, an adenine analogue substituted at the 2-position with a (4-hydroxy)-2-phenylethylamino group and bearing a furyl ring at the 8-position, was designed and synthesized together with its 4-methoxy analogue. At the 9-position of these molecules, we introduced an ethyl group that favors the interaction of adenine derivatives with A_2A_AR [31] (Figure 2a). We also replaced the furyl ring present in the 8-position of ZM241385 with an ethoxy group (Figure 2b).

Furthermore, aimed at improving the A_2A_AR binding affinity of ANR 94, we designed and synthesized its triazolotriazine analogue, which lacks, for structural requirement reasons, the ethyl group present at the 9-position of the ANR 94 purine ring (Figure 2b). 

All the newly synthesized compounds were evaluated in binding and functional studies in order to assess their affinity/activity at ARs and the compound endowed with the most promising profile of A_2A_AR affinity and selectivity was also evaluated in a microglial model of neuroinflammation.

## 2. Results and Discussion

### 2.1. Chemical Synthesis

**Scheme 1:**
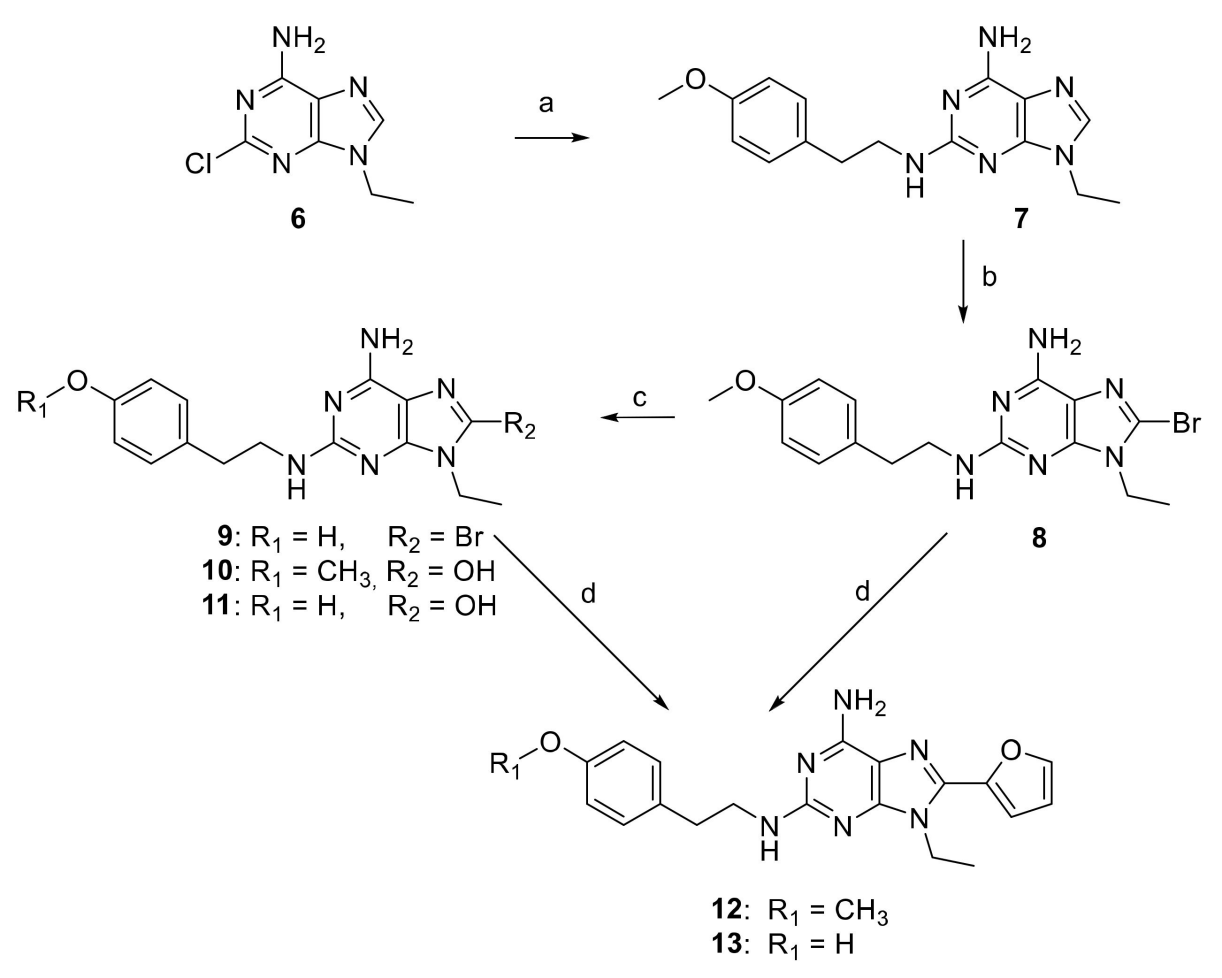
Reagents and conditions: (**a**) 4-methoxyphenethylamine at 130 °C for 24 h, 86% yield; (**b**) NBS, dry DMF, r. t. for 30 min, 44% yield; (**c**) HBr 48% at 100 °C for 2 h, 23, 10, and 32% yields for compounds **9**, **10**, **11**, respectively; (**d**) 2-tributylstannylfuran, [(Ph)_3_]_2_PdCl_2_, dry THF at reflux for 4 h (**12**, 35% yield) and 6 h (**13**, 67% yield).

The 9-ethyl-8-furyl-2-(4-hydroxyphenyl)-2-ethylaminoadenine (**13**) and its 4-methoxyphenyl analogue **12** were synthesized starting from the 2-chloro-9-ethyladenine (**6**), obtained from the commercially available 2,6-dichloropurine, in two steps [32]. Compound **6** was reacted with the p-methoxyphenethylamine, at 130 °C for 24 h, to give the disubstituted adenine derivative **7**, which was brominated using N-bromosuccinimide (NBS) in dimethylformamide (DMF) for 30 min at r. t. to obtain the 8-bromo derivative **8** (Scheme 1). The latter was treated with hydrobromic acid (48% solution in water), at r. t. for 2 h, in order to convert the phenylmethoxy group to a phenol. In this reaction, compounds **10** and **11**, deriving from the substitution of the 8-bromine atom and partial hydrolysis of the methyl ether, were obtained together with the desired 8-bromo-2-(4-hydroxy-phenyl)-2-ethylaminoadenine (**9**). Compounds **8** and **9** were, in turn, reacted with (2-tributylstannyl)furane, in tetrahydrofurane (THF) at r. t. for 16 h, to obtain the desired trisubstituted adenine derivatives **12** and **13**.

**Scheme 2:**
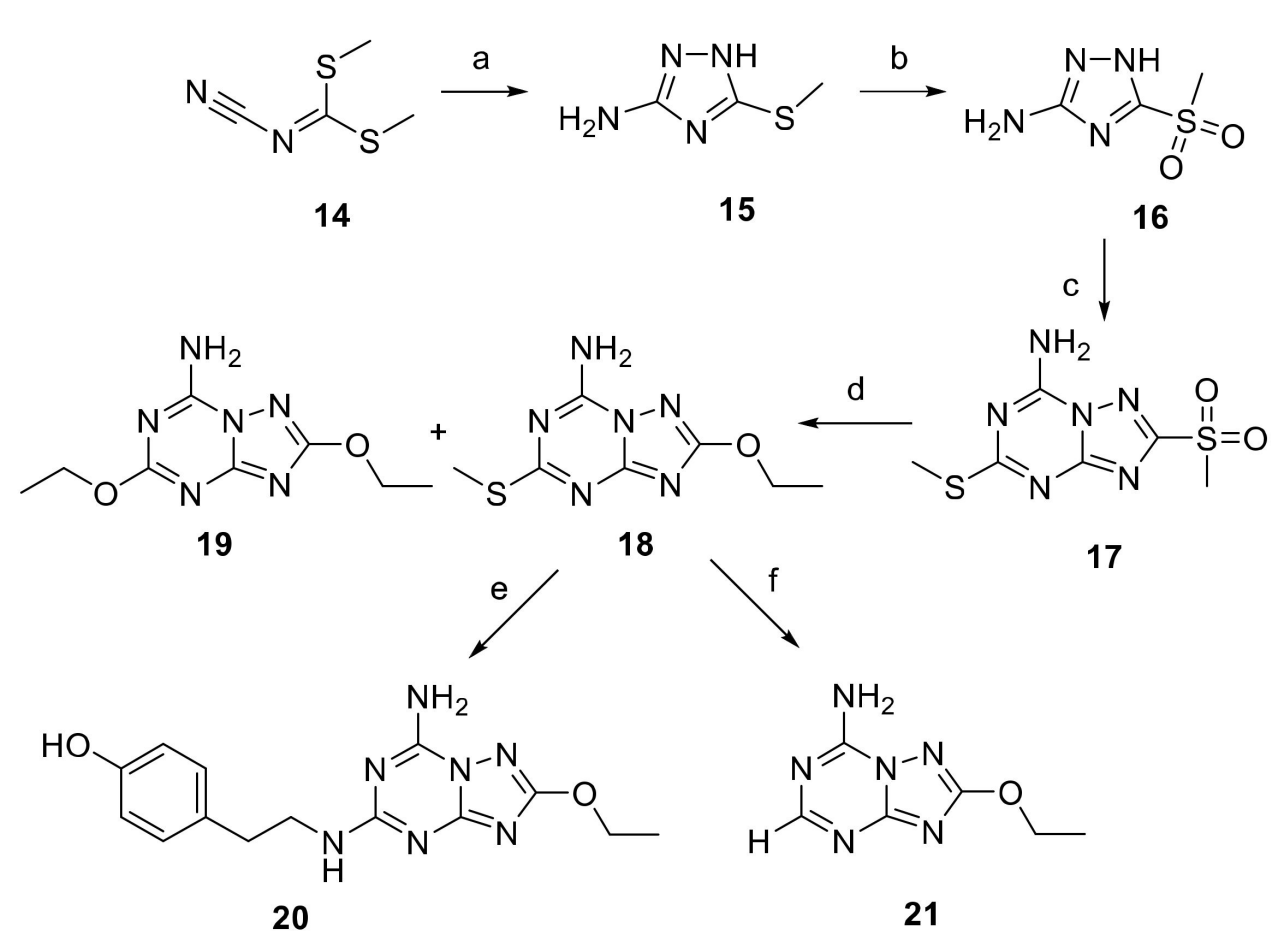
Reagents and conditions: (**a**) hydrazine hydrate, toluene, 0 °C for 1 h, r. t. for 16 h, 83% yield; (**b**) *m*-CPBA, DCM, from 0 °C to r. t., 80% yield; (**c**) **14**, 170 °C, 6.5 h, 22% yield; (**d**) EtOH, NaH, 24 h, r. t., 32 and 5% yields for compounds **18** and **19**, respectively; (**e**) *m*-CPBA, THF of 2 h, tyramine, Et_3_N, 40 °C, 12 h, 10% yield; (**f**) Et_3_SiH, Pd/C 10%, THF, 8 h, r. t., 44% yield.

For the synthesis of the new [1,2,4]-triazolo-[1,5-*a*]-[1,3,5]-triazine derivatives **20** and **21**, a targeted approach, aimed at forming first the triazole nucleus and then the triazine ring, was used (Scheme 2). A similar approach was also utilized for the synthesis of ZM241385 [33], but here we chose the commercially available dimethyl *N*-cyanodithioiminocarbonate (**14**) as the starting material which presents two very important advantages: the low price and the fact that it can be used both for the formation of the triazole and the triazine ring. In fact, **14** was reacted with aqueous hydrazine in toluene at 0 °C to afford the 5-(methylthio)-1*H*-1,2,4-triazol-3-amine (**15**). 

The formation of the triazole ring was followed by the oxidation of the **15**-thiol group, using meta-chloroperbenzoic acid (m-CPBA) in dichloromethane at r. t., to give the sulfone derivatives **16**. As already mentioned, the triazine ring was obtained by treating **16** with the starting reagent **14** in a fusion reaction performed in a steel bomb at 170 °C for 4 h. The reaction led to the formation of a main product which was isolated after chromatography on a silica gel column. Since this reaction could lead to the formation of two or more isomers of **17** [34], the structure of the obtained compound was unequivocally attributed through a further crystallographic X-ray analysis (see next section).

The reaction of **17** with sodium ethoxyde, obtained by treating ethanol with sodium hydride for 24 h, furnished the 5-ethoxy derivative **18** as the main product together with a small percentage of the disubstituted derivative **19**. 

The ZM241385 analogue **20** was obtained in a two-step reaction. First, the oxidation of the thiomethyl group of **18** with m-CPBA to the sulfone, which is a better leaving group, followed after 2 h when the starting material was totally consumed by the addition of tyramine. Then, the reaction mixture was left at 40 °C for 12 h. 

Finally, hydrogenolisis of **18**, using triethylsilane as a proton donor and Pd/C 10% as the catalyst, allowed the removal of the thiomethyl goup to obtain the desired triazolotriazine **21**.

### 2.2. Crystallographic X-ray Analysis

**Figure 3:**
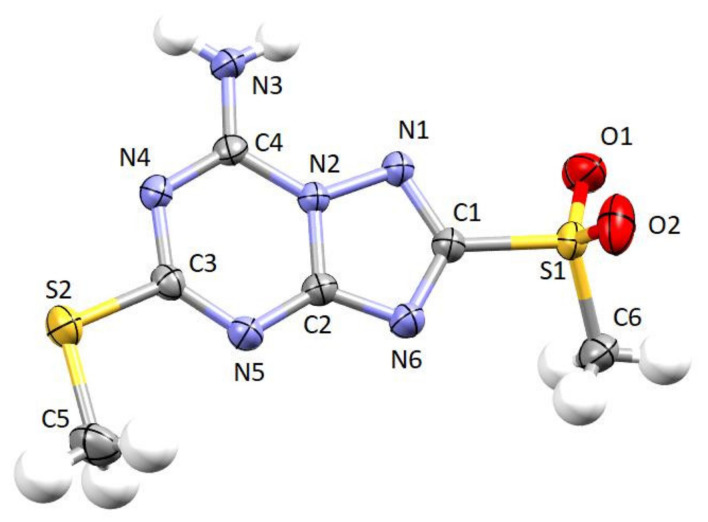
Ortep style view of compound **17**. Ellipsoids are shown at 50% probability level. Selected bond distances (Å) and angles (°): C1-N1 1.323(2), C1-N6 1.353(2), C1-S1 1.7794(16), C2-N6 1.332(2), C2-N5 1.344(2), C2-N2 1.3728(19), C3-N5 1.317(2), C3-N4 1.365(2), C3-S2 1.7480(16), C4-N3 1.311(2), C4-N4 1.325(2), C4-N2 1.3721(19), C5-S2 1.797(2), N1-N2 1.3748(17); N1-C1-N6 118.47(14), N1-C1-S1 118.59(12), N6-C1-S1 122.64(12), N6-C2-N5 129.47(14), N6-C2-N2 109.12(13), N5-C2-N2 121.41(14), N5-C3-N4 128.40(14), N5-C3-S2 120.45(12), N4-C3-S2 111.14(11), N3-C4-N4 123.52(15), N3-C4-N2 119.13(14), N4-C4-N2 117.35(14), C1-N1-N2 99.58(12), C4-N2-C2 121.84(13), C4-N2-N1 127.06(13), C2-N2-N1 110.91(12), C4-N4-C3 117.44(13), C3-N5-C2 113.48(13), C2-N6-C1 101.90(13).

The ORTEP style view of **17** is reported in Figure 3 together with the atomic labeling scheme. The most important bond distances and angles are reported in the caption. The triazolotriazine system is planar, atoms S1 and S2 are at a distance of 0.147(2) and 0.018(2) Å from the mean plane, respectively. The C-N bond distances are in the range expected for parent systems found in the literature. The crystal packing of the molecule is built up through Van der Waals and hydrogen bond interactions. In particular, N3 and N4 atoms of two adjacent molecules are involved in strong hydrogen bonds. The N3⋯N4′ distance is 3.034(2) Å, and the N3-H3A⋯N4′ angle is 174.14(9)°. Moreover, N3 is at a distance of 3.039(2) Å from O1 of another molecule in the packing. Hence, X-ray analysis unequivocally confirmed the structure of the desired triazolotriazine derivative **17**.

### 2.3. Biological Evaluation

#### 2.3.1. Binding Evaluation at ARs

The affinities of the newly synthesized compounds **7**–**13** and **17**–**21** were determined in binding studies at human recombinant A_1_, A_2A_, and A_3_ ARs, stably transfected in Chinese hamster ovary (CHO) cells, using [^3^H]CCPA (2-chloro-N^6^-cyclopentylAdo), [^3^H]NECA (5′-N-ethylcarboxamidoAdo), and [^3^H]HEMADO (2-hexynyl-N^6^-methylAdo) as respective radioligands [35,36]. Most of the compounds were also evaluated at A_2B_ARs in a functional study using the GloSensor cAMP assay [37]. The results are reported in Table 1 as Ki values in nM (± standard errors). In the case of A_2B_ARs, Ki-values were calculated from IC_50_-values determined by the inhibition of NECA-stimulated adenylyl cyclase activity. ZM241385 (**4**) and the 9-ethyl-8-ethoxyadenine (ANR 94, **5**) are reported as reference compounds. 

As already mentioned in the introduction, ZM241385 (**4**) possesses a high affinity for the A_2A_AR (Ki = 1.6 nM), good selectivity versus the A_1_ and A_3_ AR subtypes by 484- and 464-fold, respectively, and lower selectivity towards the A_2B_AR by just 47 times.

Its purine analogue maintained high affinity at the A_2A_AR but showed an inverse profile of selectivity that decreased towards the A_1_ and A_3_ subtypes and increased versus the A_2B_AR (**13**; Ki A_2A_ = 1.8 nM; selectivity A_1_/A_2A_ = 28, A_3_/A_2A_ = 7, and A_2B_/A_2A_ = 796). Supposing that the triazoletriazine scaffold of ZM241385 and the purine core of **13** are positioned with the same orientation in the binding pocket of the A_2A_AR, the presence of the ethyl group in 9-position of **13** seems to favor the interaction of this molecule with the A_1_ and A_3_ subtypes and disadvantage the one with the A_2B_AR, while it does not seem to influence the interaction with the A_2A_AR. These findings indicate that, at least with regards to A_2A_AR affinity, the triazoletriazine and purine scaffolds are interchangeable. When the phenolic hydroxyl group of **13** is replaced by a methoxy substituent, a further increase in affinity at the A_1_AR is observed, leading to an A_1_/A_2A_ unselective ligand (**12**; Ki A_2A_ = 1.0 nM and Ki A_1_ = 2.6 nM). As expected, the presence of an 8-bromine atom, instead of a furyl ring, led to a decrease in affinity at A_1_, A_2A_, and A_3_ ARs; in fact, **9** and **8** showed higher Ki values with respect to **12** and **13** while remaining A_2A_ selective ligands (**9**; Ki A_2A_ = 44 nM and **8**; Ki A_2A_ = 34 nM). The 8-unsubstituted derivative **7** lost affinity at all ARs.

These findings are in agreement with our previous results which demonstrated that the presence of an 8-bromine atom and, even more, a 8-furyl ring, favor the interaction of 2-9-disubstituted adenine derivatives at ARs [29]. Furthermore, the presence of a hydroxyl group at the 8-position of these molecules is detrimental for the affinity, especially at the A_2A_ and A_3_ subtypes (**10**; Ki A_2A_ > 30,000 nM and **11**; Ki A_2A_ and Ki A_3_ > 30,000 nM).

The 8,9-disubstituted adenine derivative ANR 94 exhibits good A_2A_AR affinity and selectivity (**5**; Ki A_2A_ = 46 nM; selectivity A_1_/A_2A_ = 52, A_3_/A_2A_ = 457, and A_2B_/A_2A_ > 652). Unfortunately, its triazolotriazine analogue **20** showed a decrease in A_2A_AR affinity by 44-fold (Ki = 2029 nM) and a decrease at the A_1_ subtype but not at the A_3_AR (Ki = 1019 nM). In this case, the replacement of the purine ring with the triazolotriazine scaffold had a negative impact on the A_2A_AR affinity. Among the 2,5-disubstituted triazoletriazines reported here, only compounds **18** and **21**, the latter being the 8-ethoxy analogue of ZM241385, showed a sub-microM affinity at the A_2A_AR with Ki values of 583 nM and 178 nM, respectively. 

The obtained results did not allow to give a univocal answer to the question of the interchangeability between the two scaffolds that we have tried to explain with molecular modeling studies.

#### 2.3.2. Study of the Anti-Inflammatory Activity of **13**

In order to investigate whether the promising affinity profile of **13** could be associated with potential protective activity against PD, we studied its anti-inflammatory properties in a microglial model of neuroinflammation. Increasing evidence indicates that neuroinflammation mediated by microglia plays a major role in PD [40,41,42]. Although it is still unclear whether the neuroimmune response may or not represent a primary cause for neuronal loss, there is wide consensus that it participates in disease progression [43]. ARs expressed in microglial cells modulate inflammatory response in PD and preliminary data suggest that A_2A_AR antagonists may have therapeutic efficacy [8,44]. 

Activated microglia can assume two different polarization states: a pro-inflammatory “M1” phenotype and an anti-inflammatory “M2” phenotype. Interestingly, in PD patients, an increase in M1-polarized microglia is observed [45]. M1-phenotype microglia is characterized by the increased production of inflammatory cytokines, such as interleukin-1β (IL-1β) and IL-6, that leads to tissue damage. On the other hand, the M2-phenotype is characterized by an upregulation of anti-inflammatory mediators, such as IL-10. In activated microglia, these two phenotypes can coexist, revealing the complexity of microglia function and the dynamic changes of the environment in vivo [46]. Different studies associate the release of pro-inflammatory mediators with the activation of the NOD-like receptor family, the pyrin domain containing-3 protein (NLRP3) inflammasome in BV-2 cells [47]. NLRP3, a multi-protein complex, modulates the maturation and secretion of pro-inflammatory cytokines, including IL-1β. Of note, the inhibition of the NLRP3 inflammasome activation could protect dopaminergic neurons [48,49]. On these bases, the identification of new compounds able to inhibit the activation of NLRP3 Inflammasome are a promising therapeutic intervention against neurodegeneration in PD.

The microglial BV-2 cell line exposed to bacterial endotoxin lipopolysaccharide (LPS) was chosen as the in vitro model of neuroinflammation. LPS, a pro-inflammatory mediator, is widely used in in vitro studies to activate microglia cells and induce proinflammatory transduction pathways [50,51,52]. In addition, BV-2 cells are a valid alternative to primary microglia as 90% of the genes regulated by LPS in BV-2 cells are also modulated in primary microglia and both display similar reaction patterns [53].

**Figure 4:**
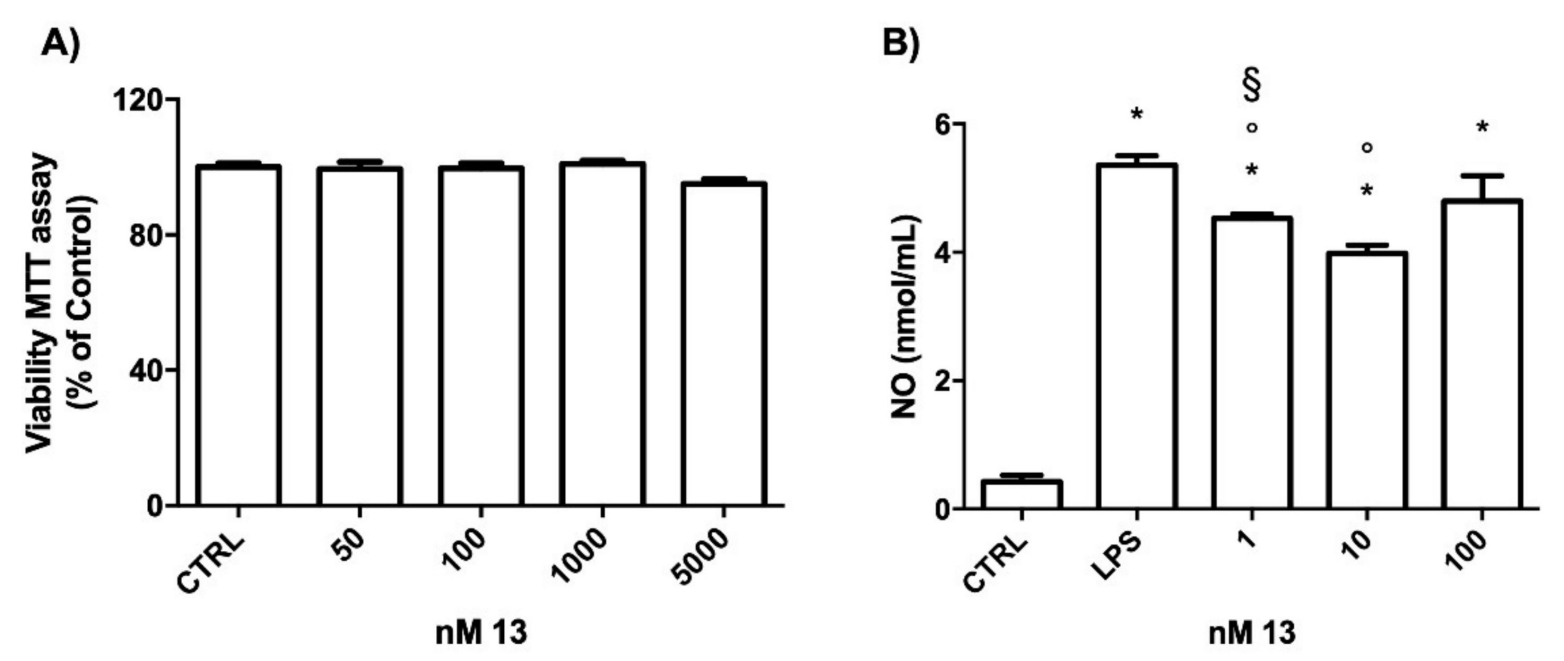
Effect of **13** on cell viability and NO production in BV-2 cells. (**A**) Cells were treated with 50–5000 nM of **13** for 24 h, and MTT assay was used to measure cell viability. (**B**) Cells were treated with 10 nM of **13** before activation with 100 ng/mL LPS for 24 h and NO release was measured by the Griess assay. Each bar represents means ± SEM of at least three independent experiments. Data were analyzed by one-way ANOVA followed by Bonferroni’s test. * *p* < 0.05 vs. CTRL; ° *p* < 0.05 vs. LPS; § *p* < 0.05 vs. 10 nM.

First of all, we verified the potential cytotoxicity of **13** in our cell model system. BV-2 cells were treated with increasing concentrations of the compound for 24 h and cell viability was evaluated by MTT assay (Figure 4A). Of note, the tested A_2A_AR antagonist did not show any cytotoxicity up to 5 μM, demonstrating its high safety profile.

To determine the effects of **13** on LPS-induced inflammatory mediators, we initially evaluated the production of NO. BV-2 cells were treated with different concentrations of **13**, then, were exposed to 100 ng/mL LPS for 24 h, and the release of NO in the culture medium was measured by Griess assay (Figure 4B). LPS strongly and significantly increased the release of NO in the culture medium with respect to the control cells (CTRL). 

The treatments with **13** at the concentrations 1 and 10 nM significantly reduced the NO level with respect to LPS treated cells. Interestingly, 10 nM treatment was the most effective in counteracting NO release as 1 nM led to significantly higher NO production than 10 nM. On theses bases, the concentration 10 nM was chosen for the following experiments. 

**Figure 5:**
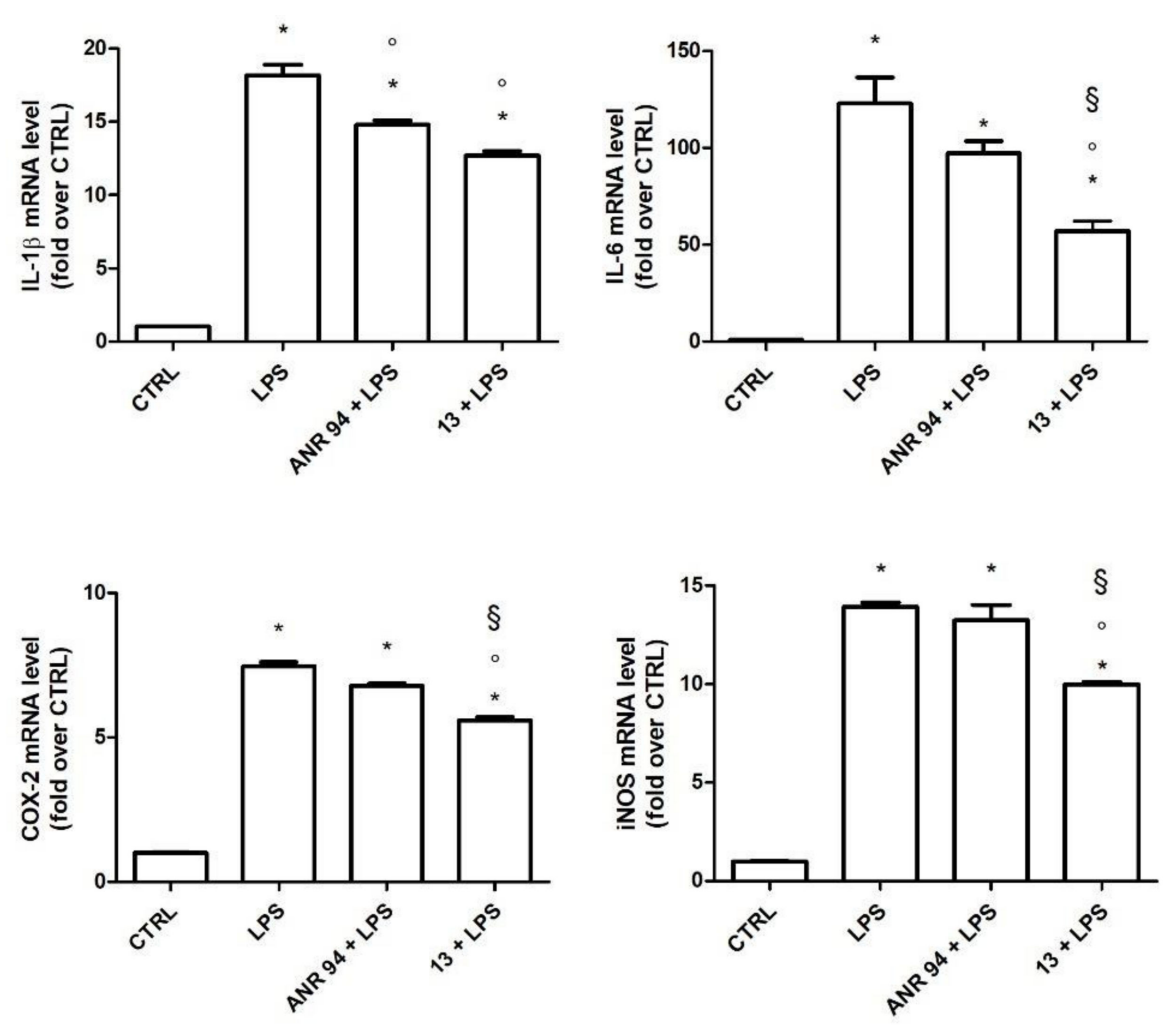
Expression of IL-1β, IL-6, COX-2, and iNOS in BV-2 cells treated with **13** and ANR 94. Cells were treated with 10 nM **13** or 10 nM ANR 94 for 24 h, exposed to 100 ng/mL LPS for 24 h and cytokines and enzymes mRNA levels were measured by RT-PCR. Data are expressed as relative abundance compared to untreated cells. Each bar represents the mean ± SEM of three independent experiments. Data were analyzed with one-way ANOVA followed by Bonferroni’s test. * *p* < 0.05 vs. CTRL; ° *p* < 0.05 vs. LPS; § *p* < 0.05 vs. ANR 94 + LPS.

To better characterize the anti-inflammatory activity of **13,** we evaluated the expression of the main pro-inflammatory cytokines and enzymes up-regulated in activated microglial cells, such as IL-1β, IL-6, inducible NO synthase (iNOS), and cyclooxygenase 2 (COX2). ANR 94 was chosen as the reference compound as we already demonstrated its ability to reduce microglia activation in a murine model of PD [8]. BV-2 cells were treated with **13** or ANR 94 (10 nM) and activated with 100 ng/mL LPS for 24 h. Total RNA was isolated, and proinflammatory cytokine and enzyme expressions were measured using RT-PCR (Figure 5). As expected, LPS significantly increased the expression of IL-1β, IL-6, COX-2, and iNOS with respect to the control cells. **13** significantly reduced the expression of all the pro-inflammatory mediators tested with respect to cells exposed to LPS. On the other hand, ANR 94 was only able to significantly reduce the expression of IL-1β. In particular, the treatment with **13** led to a significant down-regulation of IL-6, iNOS, and COX-2 with respect to ANR 94, demonstrating it possesses higher efficacy than ANR 94 in reducing the expression level of these pro-inflammatory mediators. 

**Figure 6:**
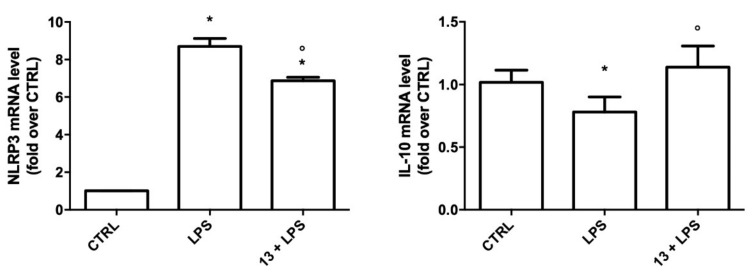
Expression of NLRP3 and IL-10 in BV-2 cells treated with **13**. Cells were treated with 10 nM **13**, exposed to 100 ng/mL LPS for 24 h and NLRP3 and IL-10 mRNA levels were measured by RT-PCR. Data are expressed as relative abundance compared to untreated cells. Each bar represents the mean ± SEM of three independent experiments. Data were analyzed with one-way ANOVA followed by Bonferroni’s test. * *p* < 0.05 vs. CTRL; ° *p* < 0.05 vs. LPS.

To verify if the ability of **13** to reduce the previous inflammatory mediators is also associated with a reduction in the inflammasome, we measured the expression of NLRP3 inflammasome (Figure 6). The data demonstrated that, after LPS stimulation, the expression of NLRP3 mRNA, which is the rate-limiting step for inflammasome activation [54], was significantly increased. Moreover, the increased mRNA of NLRP3 was significantly reduced by **13** pre-treatment. 

As IL-10 was reported to promote microglial M2 polarization [55], we then evaluated the expression of IL-10 in BV-2 cells treated with **13** before LPS activation (Figure 6). As expected, LPS significantly reduced the expression of this anti-inflammatory cytokine with respect to the control cells, while treatment with **13**, in agreement with the previous results, maintained IL-10 expression to a level comparable to the control cells, suggesting that **13** promotes the protective M2 phenotype.

In conclusion, **13** could represent a promising A_2A_AR antagonist in the fight against PD due to its ability to reduce pro-inflammatory mediators and to increase anti-inflammatory cytokines in microglia cells. Future studies are needed to increase the knowledge of its anti-inflammatory molecular mechanisms and to verify its efficacy in animal models of PD before suggesting this interesting compound for clinical applications.

### 2.4. Molecular Modeling Studies

Molecular modeling studies, consisting of docking experiments at the A_2A_AR, A_1_AR, and A_3_AR 3D structures, were performed to analyze the binding data of the developed compounds. We employed the crystal structures of the human A_2A_AR and A_1_AR in combination with ZM241385 and the antagonist PSB36, respectively, downloaded from the Protein Data Bank webpage (http://www.rcsb.org accessed on 7 January 2022; pdb code: 5NM4; 1.7-Å resolution [26], and pdb code: 5N2S; 3.6-Å resolution [56], respectively). A homology model of the human A_3_AR was built using the above cited X-ray structure of the antagonist-bound A_1_AR as a template (pdb code: 5N2S). Docking analyses were performed by using CCDC Gold [57] and then analyzed within the Molecular Operating Environment (MOE, version 2020.09) suite [58].

The docking analysis with the compound ZM241385 at the A_2A_AR was useful for checking the reliability of the docking protocols, by comparing its obtained top score docking conformation with the co-crystallized arrangement of the same molecule within the receptor structure. Results were in good agreement with the experimental data (RMSD = 1.31 Å).

**Figure 7:**
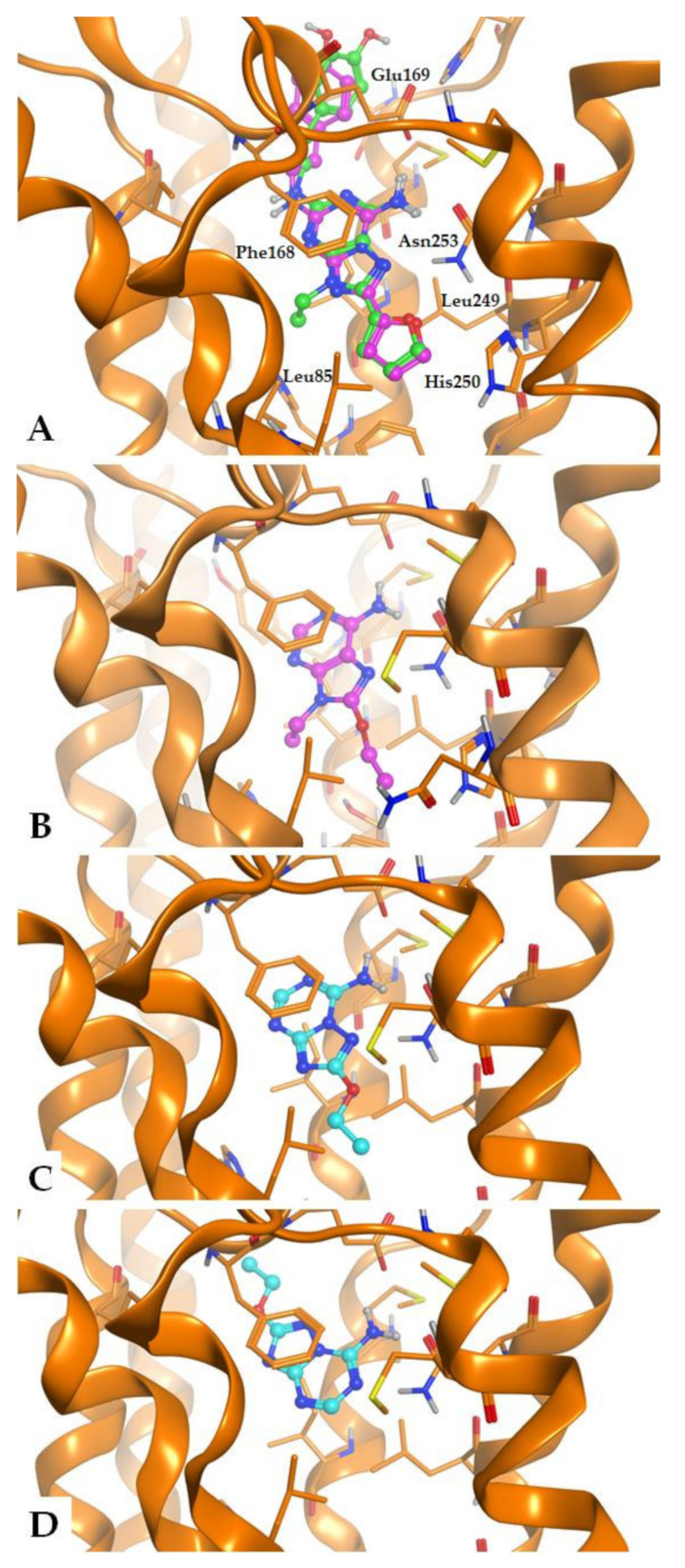
General binding mode of the synthesized compounds at the A_2A_ AR (pdb: 5NM4) binding cavity, with indication of some key receptor residues. (**A**) Compound **13** is shown in green, with the co-crystallized ligand ZM241385 (purple) for comparison; (**B**) Docking conformations of compound ANR 94 (**5**, purple); (**C**,**D**) Docking conformations of compound **21** (cyan) with representation of the two observed potential binding modes.

The docking conformations generally observed for the new adenine derivatives at the A_2A_AR are similar to those obtained in our previous studies and are showed in Figure 7A, where the purine derivative **13** is perfectly overlapped to the co-crystallized ligand ZM241385. The bicyclic purine core is positioned between the side chains of Phe168 (EL2) and Leu249^6.51^ and gives non-polar interactions with these residues. The *N*^6^-amine group makes H-bond interactions with Asn253^6.55^ and Glu169 (EL2), while the 8-substituent is located in the depth of the binding cavity. The 2-substituent is positioned at the entrance of the binding site and points toward the extracellular environment. 

Purine derivatives lacking the 2-substituent (i.e., ANR 94 (**5**), Figure 7B) or presenting a small moiety in the same position, are observed to give an analogue binding mode (respect to **13** and ZM241385), with interactions given by the purine core and the exocyclic amine group in the 6-position, and further interactions made by the other substituents. Hence, the lack of the 2-substituent or its substitution with a small group does not seems critical for the binding mode of the 9-ethyladenines. In fact, both **13** and 2-unsubstituted ANR 94 possess nM affinity at the A_2A_AR. In the case of compound **21**, based on a triazoletriazine scaffold and lacking the exocyclic ethyl chain (compared to ANR 94) and the (4-hydroxy)-2-phenylethylamino group (compared to ZM241385), we observed two potential binding modes (Figure 7C,D). 

One of these binding modes presents the triazoletriazine scaffold of **21** as almost superimposable to the one of ZM241385, while the other docking conformation makes the triazoletriazine moiety oppositely oriented (see the docking conformation colored in cyan, Figure 7C,D, respectively). We may assume that the presence of a 9-ethyl group in the adenine derivatives makes their binding mode stably anchored within the binding cavity. On the contrary, the triazolotriazine derivatives lacking the long (4-hydroxy)-2-phenylethylamino group are less stably inserted within the binding cavity, giving more potential binding modes. This feature appears to be slightly beneficial in the case of the A_3_AR, for which the affinity of **21** is higher than ANR 94.

**Figure 8:**
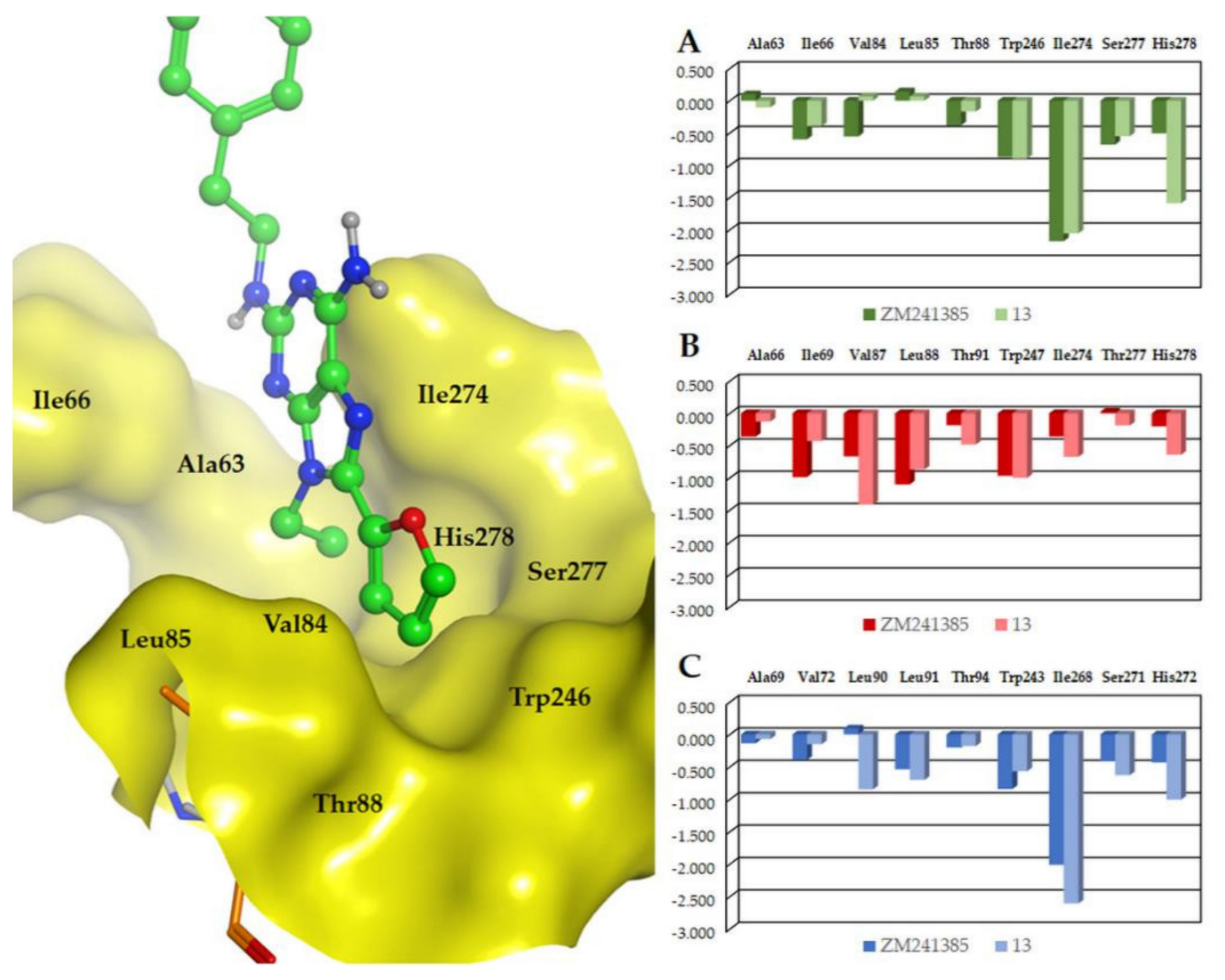
Interaction energies (represented as kcal mol^−1^) calculated with the aid of the *IF-E 6.0* tool at the A_2A_AR (**A**), A_1_AR (**B**), and A_3_AR (**C**) binding sites. Compounds ZM241385 and **13** were submitted to analysis. Receptor residues in proximity to the compound region corresponding to the 9-ethyl substituent of **13** were considered. In the panel at left, there is a representation of the analyzed residues and their position with respect to the 9-ethyl group (surface representation); the labels indicate the A_2A_AR residues. See text for details.

Compounds ZM241385 and **13** make the same interactions with the A_2A_AR, apart from the ligand region corresponding to the 9-position of the purine core. Considering the purine derivative, its 9-ethyl substituent is located in the depth of the cavity and makes hydrophobic interactions with the residues in proximity, such as Ala63^2.61^, Ile66^2.64^, Val84^3.32^, Leu85^3.33^, Thr88^3.36^, Trp246^6.48^, Ile274^7.39^, Ser277^7.42^, and His278^7.43^ (see Figure 8). Binding affinity data of ZM241385 and **13** show that at the A_2A_AR they are endowed with comparable affinity, while **13** presents higher affinity (with respect to ZM241385) at the A_1_AR and A_3_AR. Given that the unique structural difference between these two molecules is the presence of a 9-ethyl group in the purine derivative and a nitrogen atom in the corresponding position of ZM241385, this feature appears responsible for the different affinity of the above cited molecules at these two receptors. We analyzed and compared the ability of these two compounds to interact with the A_2A_AR, A_1_AR, and A_3_AR with the aid of the *IF-E 6.0* tool (see Experimental section for details), which calculates the atomic and residue interaction forces and the per-residue interaction energies (expressed as kcal × mol^−1^). This tool was previously used for analogue analyses at hARs obtaining useful interpretation of the compound activity [59,60,61]. We focused on the above residues located in proximity of the 9-ethyl group of **13**. The results are displayed in Figure 8. 

The comparison of the interaction energies calculated for ZM241385 and **13** at the A_2A_AR shows that the two compounds make analogue interactions with a sum of −5.53 and −5.63 kcal mol^−1^, respectively. This is in agreement with the similar binding affinity data of the two molecules at this AR subtype. At the A_1_AR and the A_3_AR, the two molecules show a different pharmacological behavior, with significantly higher affinity for both receptors shown by **13** compared to ZM241385. The interaction energies calculated at these receptors are again in agreement with the biological data, since at the A_1_AR and the A_3_AR the sum of the interaction energies for **13** is −5.84 and −6.76 kcal mol^−1^, respectively, while at the same receptors the sum of the interaction energies for ZM241385 is −4.82 and −4.88 kcal mol^−1^, respectively. Hence, the presence of an ethyl group in the 9-position of adenine appears beneficial to achieve affinity at the analyzed AR subtypes, while its absence appears to provide some selectivity for the A_2A_AR.

## 3. Materials and Methods

### 3.1. Chemical Synthesis

#### General Methods

Melting points were determined with a Büchi apparatus and are uncorrected. ^1^H NMR and ^13^C NMR spectra were obtained with a Bruker Ascend 500 MHz spectrometer; δ values are in ppm, *J* values are in Hz. All exchangeable protons were confirmed by the addition of D_2_O. ^13^C NMR spectra are reported in the Supplementary Materials. Mass spectra were recorded on an HP 1100-MSD series instrument. Thin-layer chromatography (TLC) was carried out on pre-coated TLC plates with silica gel 60 F_254_ (Fluka). For column chromatography, silica gel 60 (Merck) was used. Elemental analyses were determined on Fisons Instruments Model EA 1108 CHNS-O model analyzer and are within 0.4% of theoretical values. Purity of the compounds is ≥98% according to elemental analysis data.

*9-Ethyl-N^2^-(4-methoxyphenethyl)-9H-purine-2,6-diamine* (**7**): **6** (287 mg; 1.45 mmol) was suspended in 4 mL of 4-methoxyphenethylamine in a sealed vessel and was left to react at 130 °C for 24 h. Volatiles were removed under vacuum and the crude was purified by gravimetric chromatography eluted with EtOAc-*c*Hex-MeOH (60:36:4). The desired compound **7** (387 mg, 1.24 mmol; yield 86%) was obtained as white powder after crystallization by MeCN-MeOH. M.p.: 151–153 °C; ^1^H-NMR (DMSO-*d_6_*) δ: 1.38 (t, 3H, *J* = 7.3 Hz, CH_2_*CH_3_*), 2.78 (t, 2H, *J* = 7.2 Hz, CH_2_Ph), 3.41 (m, 2H, *CH_2_*NH), 3.73 (s, 3H, CH_3_O), 4.02 (q, 2H, *J* = 7.2 Hz, *CH_2_*CH_3_), 6.22 (t, 1H, NH), 6.65 (s, 2H, NH_2_), 6.87 (d, 2H, *J* = 8.3 Hz, H-Ph), 7.19 (d, 2H, *J* = 8.3 Hz, H-Ph), 7.73 (s, 1H, H-8). ESI-MS: positive mode *m/z*: 313.0 ([M+H]^+^), 335.0 ([M+Na]^+^). Elemental analysis calcd for C_16_H_20_N_6_O: C 61.52, H 6.45, N 26.90; found C 61.28, H 6.69, N 26.80.

*8-Bromo-9-ethyl-N^2^-(4-methoxyphenethyl)-9H-purine-2,6-diamine* (**8**): **7** (220 mg; 0.71 mmol) was dissolved in 5 mL of dry DMF and a solution of NBS (190 mg; 1.07 mmol) in 5 mL of dry DMF was added dropwise over 30 min in which there was the total consumption of the starting material. The mixture was evaporated to dryness under vacuum, neutralized with 1N NaOH, and extracted with ethyl acetate (3 × 20 mL). The organic phases were combined and placed under stirring with anhydrous sodium sulphate. The suspension was filtered, the filtrate was brought to dryness, and the residue purified via flash column chromatography eluting with EtOAc-*c*Hex (60:40). **8** (122 mg, 0.312 mmol, yield 44%,) was obtained as white powder after crystallization with MeCN. M.p.: 155–157 °C; ^1^H-NMR (DMSO-*d_6_*) δ: 1.30 (t, 3H, *J* = 7.1 Hz, CH_2_*CH_3_*), 2.77 (t, 2H, *J* = 8.1 Hz, CH_2_Ph), 3.41 (m, 2H, *CH_2_*NH), 3.73 (s, 3H, OCH_3_), 4.02 (q, 2H, *J* = 7.3 Hz, *CH_2_*CH_3_), 6.42 (bs, 1H, NH), 6.86 (m, 4H, H-Ph and NH_2_), 7.18 (d, 2H, *J* = 8.6 Hz, H-Ph). ESI-MS: positive mode *m/z*: 390.9 ([M+H]^+^), 414.9 ([M+Na]^+^). Elemental analysis calcd for C_16_H_19_BrN_6_O: C 49.12, H 4.89, N 21.48; found C 49.46, H 4.55, N 21.32.

*4-(2-((6-Amino-9-ethyl-8-bromo-9H-purin-2-yl)amino)ethyl)phenol* (**9**): **8** (120 mg, 0.307 mmol) was suspended in 4 mL of HBr 48% and heated at 100 °C for 2 h. The reaction was quenched with ice, neutralized at 0 °C with NH_4_OH 33%, followed by extraction with EtOAc (3 × 20 mL). The organic phases were dried with anhydrous sodium sulphate and filtered. The filtrate was brought to dryness and the residue purified via flash column chromatography eluting with EtOAc-*c*Hex-MeOH (60:39:1) to obtain **9** (44 mg, 0.12 mmol; yield 23%), **10** (19 mg, 0.06 mmol; yield 10%), and **11** (52 mg, 0.17 mmol; yield 32%). 

**9**: M.p.: 221–223 °C. ^1^H-NMR (DMSO-*d_6_*) δ: 1.19 (t, 3H, *J* = 6.6 Hz, CH_2_*CH_3_*), 2.74 (t, 2H, *J* = 7.5 Hz, CH_2_Ph), 3.32 (q, 2H, *J* = 7.0 Hz, *CH_2_*NH), 3.67 (q, 2H, *J* = 7.0 Hz, *CH_2_*CH_3_), 5.96 (bs, 2H, NH_2_), 6.25 (bs, 1H, NH), 6.80 (d, 2H, *J* = 8.4 Hz, H-Ph), 7.05 (d, 2H, *J* = 8.4 Hz, H-Ph), 9.58 (bs, 1H, OH). ESI-MS: positive mode *m/z*: 378.9 ([M+H]^+^), 400.9 ([M+Na]^+^). Elemental analysis calcd for C_15_H_17_BrN_6_O: C 47.76, H 4.54, N 22.28; found C 47.82, H 4.3, N 22.38.

**10**: m.p.: 204–205 °C. ^1^H-NMR (DMSO-*d_6_*) δ: 1.19 (t, 3H, *J* = 7.0 Hz, CH_2_CH*_3_*), 2.74 (t, 2H, *J* = 7.5 Hz, CH_2_Ph), 3.32 (q, 2H, *J* = 7.0 Hz, *CH_2_*NH), 3.67 (q, 2H, *J* = 7.0 Hz, *CH_2_*CH_3_), 3.72 (s, 3H, OCH_3_), 5.98 (bs, 2H, NH_2_), 6.21 (bs, 1H, NH), 6.85 (d, 2H, *J* = 8.4 Hz, H-Ph), 7.16 (d, 2H, *J* = 8.4 Hz, H-Ph), 9.53 (bs, 1H, OH). ESI-MS: positive mode *m/z*: 329.0 ([M+H]^+^), 351.0 ([M+Na]^+^). Elemental analysis calcd for C_16_H_21_N_6_O_2_: C 58.52, H 6.14, N 25.59; found C 58.23, H 6.43, N 25.57.

**11**: m.p.: 220 °C dec. ^1^H-NMR (DMSO-*d_6_*) δ: 1.19 (t, 3H, *J* = 7.0 Hz, CH_2_CH_3_), 2.69 (t, 2H, *J* = 7.5 Hz, CH_2_Ph), 3.67 (m, 2H, *CH_2_*NH), 4.04 (q, 2H, *J* = 7.1 Hz, *CH_2_*CH_3_), 5.99 (bs, 2H, NH_2_), 6.19 (bs, 1H, NH), 6.67 (d, 2H, *J* = 8.4 Hz, H-Ph), 7.02 (d, 2H, *J* = 8.4 Hz, H-Ph), 9.13 (bs, 1H, OH), 9.53 (bs, 1H, OH). ESI-MS: positive mode *m/z*: 315.0 ([M+H]^+^), 336.9 ([M+Na]^+^). Elemental analysis calcd for C_15_H_18_N_6_O_2_: C 57.31, H 5.77, N 26.74; found C 57.10, H 5.66, N 26.95.

General procedure for the synthesis of 4-(2-((6-Amino-9-ethyl-8-(furan-2-yl)-9H-purin-2-yl)amino)ethyl)phenol (**13**) and 9-ethyl-8-(furan-2-yl)-*N^2^*-(4-methoxyphenethyl)-9H-purine-2,6-diamine (**12**): 2-Tributylstannylfuran (104 µL, 0.33 mmol) and bis(triphenylphosphine)-palladium dichloride (3 mg, 0.004 mmol) were, in turn, added to a solution of **9** (25 mg, 0.066 mmol) or **8** (26 mg, 0.066 mmol) in anhydrous THF (330 µL). The mixtures were refluxed for 4 h and 6 h, respectively, after which volatiles were evaporated to dryness and the crude mixtures purified through a flash column silica gel eluting with DCM-MeOH (100:0 to 99:1) to afford **13** (16 mg, 0.044 mmol, yield 67%) and with DCM-MeOH (97:3) to obtain **12** (9.1 mg, 0.023 mmol, yield 35%) as white solids after crystallization from MeCN. 

**13**: m.p.: 150–151 °C. ^1^H-NMR (DMSO-d_6_) δ: 1.32 (t, *J* = 7.0 Hz, 3H, CH_2_CH_3_), 2.79 (t, *J* = 7.5 Hz, 2H, CH_2_Ph), 3.44 (q, *J* = 6.9 Hz, 2H, *CH_2_*NH), 3.72 (s, 3H, OCH_3_), 4.28 (q, *J* = 7.0 Hz, 2H, *CH_2_*CH_3_), 6.38 (bs, 1H, NH), 6.71 (m, 1H, H-4′), 6.80 (bs, 2H, NH_2_), 6.86 (d, *J* = 8.4 Hz, 2H, H-Ph), 6.98 (d, *J* = 3.3 Hz, 1H, H-3′), 7.18 (d, *J* = 8.4 Hz, 2H, H-Ph), 7.89 (m, 1H, H-5′); ESI-MS: positive mode *m/z:* 379.2 ([M+H]^+^), 391.2 ([M+Na]^+^). Elemental analysis calcd for C_20_H_22_N_6_O_2_: C 63.48, H 5.86, N 22.21; found C 63.76, H 5.58, N 22.33.

**12**: m. p.: 166–168 °C; ^1^H-NMR (DMSO-d_6_) δ: 1.29 (t, *J* = 7.2 Hz, 3H, CH_2_CH_3_), 2.71 (t, *J* = 6.8 Hz, 2H, CH_2_Ph), 3.39 (q, *J* = 6.0 Hz, 2H, *CH_2_*NH), 4.26 (q, *J* = 7.2 Hz, 2H, CH_2_CH_3_), 6.34 (m, 1H, NH), 6.66 (d, *J* = 8.0 Hz, 2H, H-Ph), 6.68 (m, 1H, H-4′), 6.79 (bs, 2H, NH_2_), 6.95 (d, *J* = 3.2 Hz, 1H, H-3′), 7.03 (d, *J* = 8.4 Hz, 2H, H-Ph), 7.87 (m, 1H, H-5′), 9.13 (s, 1H, OH); ESI-MS: positive mode *m/z:* 365.2 ([M+H]^+^), 387.2 ([M+Na]^+^); negative mode *m/z* 363.1 ([M−H]^−^), 409.1 ([M-Cl]^−^). Elemental analysis calcd for C_19_H_20_N_6_O_2_: C 62.62, H 5.53, N 23.06; found C 62.86, H 5.40, N 23.20).

*5-(Methylthio)-1H-1,2,4-triazol-3-amine* (**15**): Dimethyl *N*-cyanodithioimino-carbonate (**14**, 1.008 g, 6.89 mmol) was dissolved in toluene (3 mL) and reacted with hydrazine hydrate (228 μL, 10.33 mmol) under stirring in an ice bath under an inert atmosphere. The mixture was left at 0 °C for 1 h and then at r. t. for 4 h; after that it was filtered, and the residue was washed with *c*Hex. The organic layers were dried over sodium sulfate, then filtered and evaporated and the residue was purified by flash column chromatography eluting with DCM-MeOH (97.5:2.5) to obtain the desired compound **15** as a white solid (741 mg; 5.70 mmol; yield 83%). M.p.: 136–139 °C. ^1^H-NMR (DMSO-*d*_6_) δ: 2.38 (s, 3H, CH_3_), 5.99 (bs, 2H, NH_2_), 11.91 ppm (bs, 1H, NH). ESI-MS: positive mode *m/z*: 131.0 ([M+H]^+^), 153.0 ([M+Na]^+^). Elemental analysis calcd for C_3_H_6_N_4_S: C 27.68, H 4.65, N 43.04; found C 27.86, H 4.55, N 43.18.

*5-(Methylsulfonyl)-1H-1,2,4-triazol-3-amine* (**16**): Compound **16** was obtained by reacting the intermediate **15** (460 mg, 3.53 mmol) with *m*-CPBA (2.434 g, 14.14 mmol), both dissolved in DCM (32 mL) at 0 °C for 4 h and subsequently at r. t. for 20 h. After removal of the solvent under vacuum, compound **16** was obtained by crystallization with EtOAc, from the crude mixture, as a white solid (457 mg, 2.82 mmol, yield 80%). M.p.: 235–237 °C. ^1^H-NMR (DMSO-*d*_6_) δ: 3.18 (s, 3H, SCH_3_), 6,55 (s, 2H, NH_2_), 12.86 ppm (s, 1H, NH). ESI-MS: positive mode *m/z*: 185.0 ([M+Na]^+^), 346.9 ([2M+Na]^+^); negative mode *m/z*: 161.1 ([M−H]^−^), 197.1 ([M+Cl]^−^), 323.0 ([2M−H]^−^), 358.9 ([2M+Cl]^−^). Elemental analysis calcd for C_3_H_6_N_4_O_2_S: C 22.22, H 3.73, N 34.55; found C 22.48, H 3.47, N 34.50).

*2-(Methylsulfonyl)-5-(methylthio)-[1,2,4]triazolo [1,5-a][1,3,5]triazin-7-amine* (**17**): The intermediate **16** (1.114 g, 6.87 mmol) was reacted with dimethyl *N*-cyanodithioiminocarbonate (**14**, 2.009 g, 13.74 mmol) in a metal container hermetically sealed and placed at 170 ° C for 6.5 h. The product becomes a sticky yellow paste which was purified by flash column chromatography eluting with DCM-MeOH (99.5:0.5) to obtain the desired product **17** as a white solid (391 mg, 1.5 mmol; yield 22%). M.p.: 250 °C (dec). ^1^H-NMR (DMSO-*d*_6_) δ: 2.48 (s, 3H, SCH_3_), 3.40 (s, 3H, SO_2_CH_3_), 9.15 ppm (bs, 2H, NH_2_). ESI-MS: positive mode *m/z*: 282.9 ([M+Na]^+^), 542.6 ([2M+Na]^+^); negative mode *m/z*: 259.1 ([M−H]^−^). Elemental analysis calcd for C_6_H_8_N_6_O_2_S_2_: C 27.69, H 3.10, N 32.29; found C 27.36, H 3.23, N 32.49).

*2-Ethoxy-5-(methylthio)-[1,2,4]triazolo[1,5-a][1,3,5]triazin-7-amine* (**18**) *and 2,5-diethoxy-[1,2,4]triazolo[1,5-a][1,3,5]triazin-7-amine* (**19**): 3 mL of ethanol are reacted with sodium hydride (252 mg, 6.45 mmol) for 2 h at room temperature under nitrogen flow. Then compound **17** (150 mg, 0.61 mmol) was added and the reaction mixture was left at 50 °C for 28 h. Then, the reaction was neutralized with an aqueous solution of 2N HCl and brought to dryness under vacuum. Water was added, and the product was extracted with ethyl acetate (3 × 20 mL). The organic phases were dried with anhydrous sodium sulphate and filtered. The filtrate is brought to dryness and the residue purified via flash column chromatography eluting with DCM-MeOH (99.5: 0.5) to obtain the desired compound **18** (44 mg, 0.19 mmol; yield 32%) and a secondary product **19** as white solid (7 mg, 0.031 mmol; 5%).

**18**: m.p.: 220 °C (dec). ^1^H-NMR (DMSO-*d*_6_) δ: 1.34 (t, 3H, *J* = 7.1 Hz, CH_3_), 2.45 (s, 3H, SCH_3_), 4.36 (q, 2H, *J =* 7.0 Hz, OCH_2_), 8.33 (bs, 1H, *H*NH), 8.75 ppm (bs, 1H, *H*NH). ESI-MS: positive mode *m/z*: 227.1 ([M+H]^+^), 249.1 ([M+Na]^+^), 475.0 ([2M+Na]^+^); negative mode *m/z*: 225.3 ([M−H]^−^). elemental analysis calcd for C_7_H_10_N_6_OS: C 7.16, H 4.45, N 37.14; found C 7.46, H 4.24, N 37.00.

**19**: m.p.: 180–181 °C. ^1^H-NMR (DMSO-*d*_6_) δ: 1.27 (t, 3H, *J* = 6.8 Hz, CH_3_), 1.33 (t, 3H, *J* = 6.8 Hz, CH_3_), 4.28 (q, 2H, *J =* 7.2 Hz, OCH_2_), 4.34 (q, 2H, *J =* 7.2 Hz, OCH_2_), 8.42 (bs, 2H, NH_2_). ESI-MS: positive mode *m/z*: 225.1 ([M+H]^+^), 247.1 ([M+Na]^+^), 471.0 ([2M+Na]^+^); negative mode *m/z*: 223.2 ([M−H]^−^). Elemental analysis calcd for C_8_H_12_N_6_O_2_: C 42.85, H 5.39, N 37.48; found C 42.75, H 5.51, N 37.40.

*2-Ethoxy-[1,2,4]triazolo[1,5-a][1,3,5]triazin-7-amine* (**21**): Compound **18** (35 mg, 0.15 mmol) was suspended in tetrahydrofuran (THF, 7 mL) and Pd/C 10% (8 mg, 0.076 mmol) was added. The mixture was placed at 0 °C and then triethylsilane (159 mg, 1.37 mmol) was added. The reaction mixture was left at 0 °C for 30 min and then at r. t. for 7.5 h. The formed suspension was filtered, the filtrate was evaporated to dryness under vacuum, and the residue was crystallized from a mixture of DCM-MeOH (9:1) to obtain the desired product (12 mg, 0.066 mmol; yield 44%). M.p.: 212–213 °C. ^1^H-NMR (DMSO-*d*_6_) δ: 1.35 (t, 3H, *J* = 6.8 Hz, CH_2_*CH_3_*), 4.40 (q, 2H, *J* = 6.8 Hz, *CH_2_*CH_3_), 8.21 (s, 1H, H-5), 8.40 (bs, 1H, *H*NH), 8.85 (s, 1H, *H*NH). ESI-MS: positive mode *m/z*: 181.2 ([M+H]^+^), 203.1 ([M+Na]^+^), 383.0 ([2M+Na]^+^); negative mode *m/z*: 179.3 ([M−H]^−^), 259.2 ([2M−H]^−^). Elemental analysis calcd for C_14_H_17_N_7_O_2_: C 53.32, H 5.43, N 31.09; found C 53.16, H 5.59, N 31.20).

*4-(2-((7-amino-2-ethoxy-[1,2,4]triazolo[1,5-a][1,3,5]triazin-5-yl)amino)ethyl)phenol* (**20**): To a suspension of **18** (80 mg, 0.35 mmol) in THF, m-CPBA (88 mg, 0.51 mmol) was added at r. t. and left to react for 2 h. Then, to the reaction mixture, a solution of tyramine (77 mg, 0.35 mmol) in THF was added. The temperature was brought to 40 °C and left for 24 h. To the reaction mixture, 2N NaHCO_3_ was added to neutralize the pH and extracted with EtOAc (3 × 20 mL). The organic phases were dried with anhydrous sodium sulphate and filtered. The filtrate was brought to dryness and the residue purified via flash column chromatography eluting with DCM-MeOH (98:2). The compound was obtained by crystallization with MeOH-EtOAc-Petroleum ether (9:1:1) to obtain **20** as a white powder (4 mg, mmol; yield 10%). ^1^H-NMR (DMSO-*d*_6_) δ: 1.31 (t, 3H, CH_3_), 2.66 (m, 2H, CH_2_Ph), 3.32 (m, C*H*_2_NH_2_), 4.29 (q, 2H, OCH_2_), 6.98 (d, 2H, *J =* 7.0 Hz, H-Ph), 6.65 (d, 2H, H-Ph), 7.28 (m, 1H, NH), 9.15 (s, 1H, *H*O-Ph). ESI-MS: positive mode *m/z*: 316.1 ([M+H]^+^), 337.9 ([M+Na]^+^), 653.0 ([2M+Na]^+^). Elemental analysis calcd for C_6_H_8_N_6_O: C 40.00, H 4.48, N 46.65; found C 40.24, H 4.44, N 46.42.

### 3.2. Crystallographic Study

The crystallographic data for complex **17** were collected on a Bruker APEX II single-crystal diffractometer working with monochromatic Mo-Kα radiation and equipped with an area detector. The structure was solved by direct methods and refined against F^2^ with SHELXL-2014/7 with anisotropic thermal parameters for all non-hydrogen atoms [62,63]. Idealized geometries were assigned to the hydrogen atoms. Crystallographic data were deposited with the Cambridge Crystallographic Data Centre as a supplementary publication (reference code CCDC 2133489). Copies of the data can be obtained free of charge upon application to the CCDC, 12 Union Road, Cambridge CB2 1EZ, UK (fax, (+44) 1223 336033; e-mail, deposit@ccdc.cam.ac.uk).

### 3.3. Biological Assays

#### 3.3.1. Binding Evaluation at ARs

Cell culture: The Chinese hamster ovary cells (CHO) stably transfected with the desired ARs were grown in DMEM/F12 medium containing 10% FBS (fetal bovine serum), 100 U/mL penicillin, 100 µg/mL streptomycin, 2.5 μg/mL amphotericin B, 1 mM sodium pyruvate, 0.1 mg/mL genetecina (G418), and incubated at 37 °C in a gaseous mixture of 5% CO_2_: 95% O_2_.

Preparation of membranes: Cell membranes for binding assays were prepared mechanically detaching the cells from the petri and suspending them in a cold hypotonic buffer (5 mM Tris/HCl, 2 mM EDTA, pH 7.4). The cell suspension was homogenized (Ultra-Turrax, 2 × 15 s at maximum speed) and the homogenate centrifuged for 10 min (4 °C) to 1000× *g*. The supernatant was subsequently centrifuged for 30 min (4 °C) at 100,000× *g* and the pellet containing the membrane proteins was re-suspended in a specific buffer for each receptor subtype (A_1_: 50 mM Tris/HCl, pH 7.4; A_2A_: 50 mM Tris/HCl, 10 mM MgCl_2_, pH 7.4; A_3_: 50 mM Tris/HCl, 10 mM MgCl_2_, 1 mM EDTA, pH 8.25). The amount of the protein suspension was determined according to the Bradford method using the BCA Protein Assay Kit (Pierce), frozen in liquid nitrogen, and stored at −80 °C.

Binding assays: The dissociation constants of the radioligand (KD) were calculated through saturation assays, while the dissociation constants of the compounds under study (*K*i) were determined by competition experiments. All binding data were calculated by nonlinear regression curves using the software GraphPad Prism 5 (GraphPad Software, San Diego, CA, USA). The increasing concentrations of the radioligand saturation binding ([^3^H]-CCPA for the A_1_ receptor subtype, [^3^H]-NECA for the A_2A_ receptor subtype, and [^3^H]-HEMADO for the A_3_ receptor) subtype were incubated in a total volume of 250 μL containing 0.2 U/mL of Ado deaminase (ADA) and 12 micrograms of membrane proteins in the specific buffer. In competition experiments for the A_1_ receptor subtype, each compound was added at increasing concentrations, to the well containing 1 nM of [^3^H]-CCPA, to a final volume of 200 μL. The samples were left to incubate for 3 h at room temperature, filtered in a filter 96-well plate (UniFilter GF/C, Perkin-Elmer Life and Analytical Science, Boston, MA) using the FilterMate Cell Harvester (Perkin-Elmer), and washed 3 times with cold distilled water. After drying the filter plate at 40 °C for 30′, they 20 μL of scintillating liquid (Microscint-20, Perkin-Elmer) was added to each well, and then the radioactivity was quantized with the MicroBeta2 Plate Counter (Perkin-Elmer). The conditions for binding experiments at A_2A_ and A_3_ receptor subtypes were essentially the same as for the A_1_AR using [^3^H]-NECA (N-ethylcarboxyamideAdo) at a concentration of 10 nM and [^3^H]-HEMADO (2-(1-hexyne)-*N*^6^-methylAdo) at a concentration of 1 nM as radioligands, respectively. The samples were incubated with 12 micrograms of protein for 3 h at room temperature and filtered as described above.

The non-specific binding was determined in the presence of 1 mM theophylline for the A_1_AR, or 100 µM R-PIA (*N*^6^-(1-methyl-2-phenylethyl)Ado) for the A_2A_ and A_3_ receptors.

#### 3.3.2. Anti-Inflammatory Activity Assays

Cell Cultures and Treatments: BV-2 murine microglial cells (a gift from Prof. Elisabetta Blasi of the University of Modena and Reggio Emilia, Modena, Italy) were maintained at 37 °C and 5% CO_2_ in a humidified incubator and grown in DMEM supplemented with 10% (*v*/*v*) low-endotoxin fetal bovine serum (Euroclone—Milan, Italy), 2 mM L-glutamine, 50 U/mL penicillin, and 50 μg/mL streptomycin (Sigma-Aldrich–Merck, Milan, Italy). Cells were seeded at 50,000 cells/mL in 96-well tissue culture plates for the viability assay, in 6-well tissue culture plates for the Griess assay and RT-PCR assay. BV-2 cells were treated for 24 h with ANR 94 or **13** at different concentrations and then exposed to LPS (100 ng/mL; Sigma-Aldrich–Merck) for a further 24 h.

MTT Viability Assay: MTT assay was performed to quantify the viability of BV-2 cells as previously reported [52]. At the end of the experiments, a cell culture medium was replaced with 0.5 mg/mL MTT solution (Sigma-Aldrich–Merck), and after 30 min the formed formazan crystals were solubilized, replacing the MTT solution with DMSO. The absorbance was measured at 595 nm using a microplate spectrophotometer (VICTOR3 V Multilabel Counter; PerkinElmer, Wellesley, MA, USA). Data are expressed as a percentage of the control cells, which is considered 100% cell viability.

Measurement of NO Production: The amount of nitrite accumulation in the culture medium of BV-2 cells was measured using Griess reagent. 50 µL of culture medium from each sample was mixed with the same volume of the Griess reagent (Sigma-Aldrich-Merck). The NO concentration was evaluated by measuring the absorbance at 540 nm with a 96-well microplate spectrophotometer (VICTOR3 V Multilabel Counter; PerkinElmer). Concentration of nitrites released in the medium was determined from the standard curve generated with known concentrations of sodium nitrite. Results are expressed as mean concentration of nitrites (μM) ± SEM, from three different samples.

Real-Time Polymerase Chain Reaction (PCR): At the end of the experiments, total RNA was extracted by BV-2 cells using RNeasy Mini Kit (QIAGEN GmbH, Hilden, Germany). RNA concentraion and quality were measured on a NanoVue Spectrophotometer (GE Healthcare, Milano, Italy). iScript cDNA Synthesis Kit (Bio-Rad, Hercules, CA, USA) was used to synthesize cDNA starting from the extracted RNA, following the supplier’s instructions. PCR was performed by adding 2.5 μL (12.5 ng) of cDNA, 5 μL SsoAdvanced Universal SYBR Green Supermix (Bio-Rad), and 0.5 μL (500 nM) of each primer (Table 2) to a PCR tube. cDNA amplification was started at 95 °C for 30 s to activate the polymerase, followed by 40 cycles of 5 s at 95 °C and 30 s at 60 °C. Normalized expression levels were calculated relative to the control cells according to the 2^−ΔΔCT^ method.

### 3.4. Molecular Modeling

Receptor refinement and energy minimization tasks were carried out using MOE. Docking experiments were performed with CCDC Gold [57].

A_2A_AR and A_1_AR crystal structures refinement: The crystal structures of the human A_2A_AR and A_1_AR in complex with ZM241385 and the antagonist PSB36, respectively, were downloaded by the Protein Data Bank webpage (http://www.rcsb.org accessed on 7 January 2022; pdb code: 5NM4; 1.7-Å resolution [26], and pdb code: 5N2S; 3.6-Å resolution [56], respectively). The two structures were checked into MOE by restoring missing loops and the wild-type receptor sequences, and by adding all the hydrogen atoms. The protein structures were then energetically minimized with MOE using the AMBER99 force field until the RMS gradient of the potential energy was less than 0.05 kJ mol^−1^ Å^−1^.

Homology modeling of the human A_3_AR structure: A homology model of the human A_3_AR was built using the above cited X-ray structure of the antagonist-bound A_1_AR as a template (pdb code: 5N2S). A multiple alignment of the AR primary sequences was built within MOE as the preliminary step. The Homology Modeling tool of MOE was employed for this task. The obtained A_3_AR model was then energetically minimized with the same protocol of the other receptors (see above).

Molecular docking analysis: Docking analyses were performed by using CCDC Gold [57], which was set with default efficiency settings by selecting ChemScore as the scoring function and generating 50 poses for each ligand.

Post Docking analysis. Residue interaction analysis: The interactions between the ligands and the AR receptors binding sites were analyzed by using the *IF-E 6.0* tool retrievable at the SVL exchange service (Chemical Computing Group, Inc. SVL exchange: http://svl.chemcomp.com accessed on 7 January 2022). The program calculates and displays the atomic and residue interaction forces as 3D vectors. It also calculates the per-residue interaction energies, where negative and positive energy values are associated to favorable and unfavorable interactions, respectively. For each AR structure, a shell of residues contained within a 10 Å distance from the ligand were considered for this analysis.

## 4. Conclusions

On the base of ZM241385 and ANR 94 structures, two series of new compounds endowed with the triazolotriazine, and purine scaffolds were designed and synthesized. Radioligand binding and functional assays at human recombinant ARs showed that the 9-ethyl-8-furyl-2-(4-hydroxyphenyl)-2-ethylaminoadenine (**13**) and its 4-methoxyphenyl analogue **12** showed high affinity (Ki in the low nM range) and a different degree of selectivity at the A_2A_AR. Furthermore, **13** was found to exert anti-inflammatory properties in a microglial model of neuroinflammation, since it was able to reduce pro-inflammatory mediators and to increase anti-inflammatory cytokines in microglia cells.

Comparing the ARs binding mode of selected compounds belonging to the two series, molecular modeling studies support the hypothesis that the triazolotriazine and the purine scaffolds are interchangeable only when the 2 and 5 positions of the triazolotriazine moiety (corresponding to the purine 2- and 8-positions) are substituted. Taken together, the results of our experiments reveal that the novel A_2A_AR antagonist **13** is a potential tool for studying neuroinflammation and neuroprotective processes and could be a useful anti-PD agent suitable for further investigations.

## Figures and Tables

**Table 1:** Binding data at A_1_, A_2A_, and A_3_ ARs or adenylate cyclase activity at A_2B_AR of compounds **4**, **5**, **7**–**13**, **17**–**21** at human ARs stably transfected in CHO cells (Ki, nM).

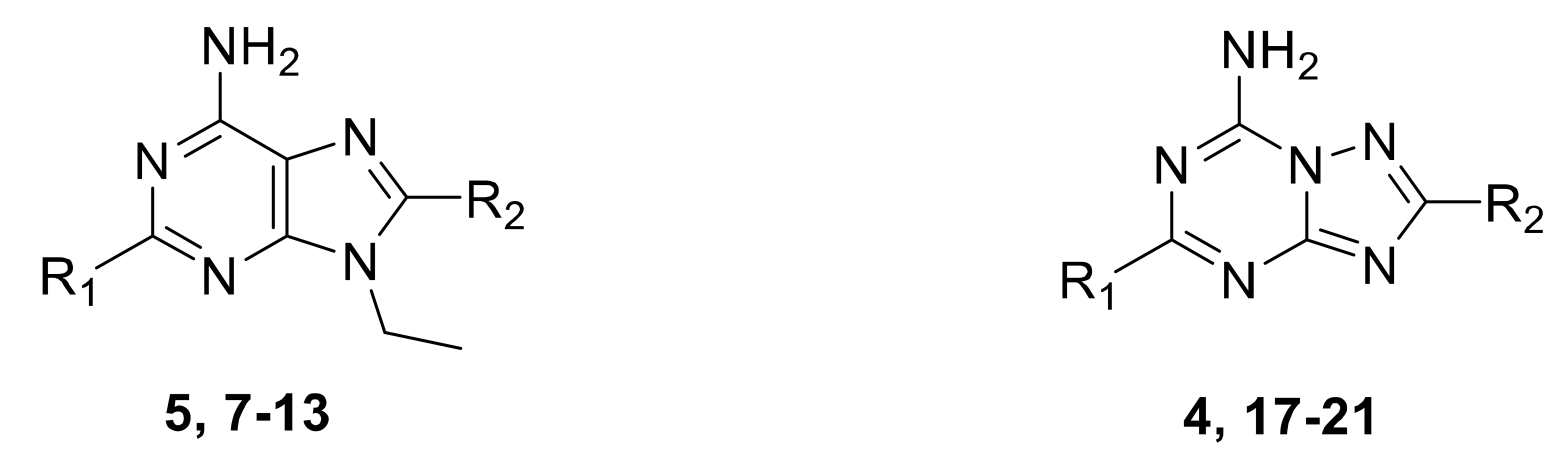						
Cpd	R_1_	R_2_	A_1_AR ^1^	A_2A_AR ^2^	A_2B_AR ^3^	A_3_AR ^4^
ZM241385 (**4**)	*p*HO-Ph(CH_2_)_2_NH	2-Furyl	774 [38]	1.6	75	743 [38]
ANR 94 (**5**)	H	OCH_2_CH_3_	2400	46	>30,000	21,000 [39]
**7**	*pC*H_3_O-Ph(CH_2_)_2_NH	H	5840 (±558)	1460 (±255)	>30,000	23,600 (±3500)
**8**	*pC*H_3_O-Ph(CH_2_)_2_NH	Br	545 (±114)	34 (±7.6)	4260 (±1795)	4030 (±825)
**9**	*p*HO-Ph(CH_2_)_2_NH	Br	411 (±104)	44 (±7.03)	393 (±133)	3810 (±205)
**10**	*pC*H_3_O-Ph(CH_2_)_2_NH	OH	1390 (±280)	>30,000	>30,000	777 (±578)
**11**	*p*HO-Ph(CH_2_)_2_NH	OH	7054 (±419)	>30,000	>30,000	>30,000
**12**	*pC*H_3_O-Ph(CH_2_)_2_NH	2-Furyl	2.6 (±0.34)	1.0 (±0.06)	>30,000	16 (±5.02)
**13**	*p*HO-Ph(CH_2_)_2_NH	2-Furyl	51 (±11)	1.8 (±0.01)	1434 (±728)	12 (±2.1)
**17**	SCH_3_	SO_2_CH_3_	5720 (±318)	3238 (±743)	n.d. ^5^	14,710 (±1600)
**18**	SCH_3_	OCH_2_CH_3_	4568 (±953)	583 (±88)	n.d. ^5^	9716 (±775)
**19**	OCH_2_CH_3_	OCH_2_CH_3_	6360 (±1298)	1061 (±217)	n.d. ^5^	4786 (±929)
**20**	H	OCH_2_CH_3_	9769 (±185)	2029 (±477)	>30,000	1019 (±172)
**21**	*p*HO-Ph(CH_2_)_2_NH	OCH_2_CH_3_	5507 (±364)	178 (±49)	>30,000	6130 (±190)

^1^ Displacement of specific [^3^H]CCPA binding in membranes from CHO cells, stably transfected with human recombinant A_1_AR. ^2^ Displacement of specific [^3^H]NECA binding in membranes from CHO cells, stably transfected with human recombinant A_2A_AR. ^3^ At A_2B_AR Ki-values were calculated from IC_50_-values determined by the inhibition of NECA-stimulated adenylyl cyclase activity. ^4^ Displacement of specific [^3^H]HEMADO binding in membranes from CHO cells, stably transfected with human recombinant A_3_AR. Data (*n* = 3–5) are expressed as means ± standard errors. ^5^ Not determined.

**Table 2:** Reports the primers used (Sigma-Aldrich–Merck). GAPDH was used as the reference gene.

Gene	5′-Forward-3′	5′-Reverse-3′
IL-1β	GTTCCCATTAGACAACTGCACTACAG	GTCGTTGCTTGGTTCTCCTTGTA
IL-6	TCCTTCAGAGAGATACAGAAAC	TTCTGTGACTCCAGCTTATC
COX-2	TGGGGTGATGAGCAACTATT	AAGGAGCTCTGGGTCAAACT
iNOS	CCTCCTCCACCCTACCAAGT	CACCCAAAGTGCTTCAGTCA
NLRP3	GATGCTGGAATTAGACAACTG	GTACATTTCACCCAACTGTAG
IL-10	CAGGACTTTAAGGGTTACTTG	ATTTTCACAGGGGAGAAATC
GAPDH	ACCACAGTCCATGCCATCAC	TCCACCACCCTGTTGCTGTA

## Data Availability

Not applicable.

## Supplementary Materials

The following supporting information can be downloaded at: https://www.mdpi.com/article/10.3390/molecules27082386/s1, ^1^H NMR and ^13^C NMR spectra of compounds **7**–**13** and **17**–**21**.
