# Supplementary material for: A2A Adenosine Receptor Antagonists: Are Triazolotriazine and Purine Scaffolds Interchangeable?
Source: Molecules. 2022 Apr 7;27(8):2386. doi: 10.3390/molecules27082386 (PMC9032385; doi:10.3390/molecules27082386)
Supplement: Supplementary file 1 [file molecules-27-02386-s001.zip › molecules-1652445-supplementary.pdf]

molecules

MDPI

Supporting Information

A_{2A} Adenosine Receptor Antagonists: Are Triazolotriazine and Purine Scaffolds Interchangeable?

Andrea Spinaci [1,†], Catia Lambertucci [1,†], Michela Buccioni [1], Diego Dal Ben [1], Claudia Graiff [2], Maria Cristina Barbalace [3], Silvana Hrelia [3], Cristina Angeloni [3], Seyed Khosrow Tayebati [4], Massimo Ubaldi [4], Alessio Masi [5], Karl-Norbert Klotz [6], Rosaria Volpini [1,*] and Gabriella Marucci [1]

1 Medicinal Chemistry Unit, School of Pharmacy, University of Camerino, Via Madonna delle Carceri, 62032 Camerino, Italy; andrea.spinaci@unicam.it (A.S.); catia.lambertucci@unicam.it (C.L.); michela.buccioni@unicam.it (M.B.); diego.dalben@unicam.it (D.D.B.); gabriella.marucci@unicam.it (G.M.)
2 Department of Chemistry, Life Sciences and Environmental Sustainability, University of Parma, Parco Area delle Scienze, 17/A, 43124 Parma, Italy; claudia.graiff@unipr.it
3 Department for Life Quality Studies, Alma Mater Studiorum, University of Bologna, Corso d'Augusto 237, 47921 Rimini, Italy; maria.barbalace2@unibo.it (M.C.B.); silvana.hrelia@unibo.it (S.H.); cristina.angeloni@unibo.it (C.A.)
4 Pharmacology Unit, School of Pharmacy, University of Camerino, Via Madonna delle Carceri, 62032 Camerino, Italy; khosrow.tayebati@unicam.it (S.K.T.); massimo.ubaldi@unicam.it (M.U.)
5 Department of Neuroscience, Psychology, Drug Research and Child's Health, NEUROFARBA, Università di Firenze, 50139 Firenze, Italy; alessio.masi@unifi.it
6 Institut für Pharmakologie und Toxikologie, University of Würzburg, Versbacher Straße 9, D-97078 Würzburg, Germany; klotz@toxi.uni-wuerzburg.de
* Correspondence: rosaria.volpini@unicam.it; Tel.: +39-07-3740-2278
† These authors contributed equally to this work.

Table of Contents

Materials and methods NMR spectroscopy--S1
^{1}H NMR and ^{13}C NMR spectra of compounds **7–13** and **18–21**--------------------------------S2

Materials and methods NMR spectroscopy

^{1}H NMR and ^{13}C NMR spectra were obtained with a Bruker Ascend 500 MHz spectrometer; δ values are in ppm, *J* values are in Hz. All exchangeable protons were confirmed by the addition of D_2O.

¹H-NMR/¹³C-NMR spectra of compounds 7–13, 18–21:

7

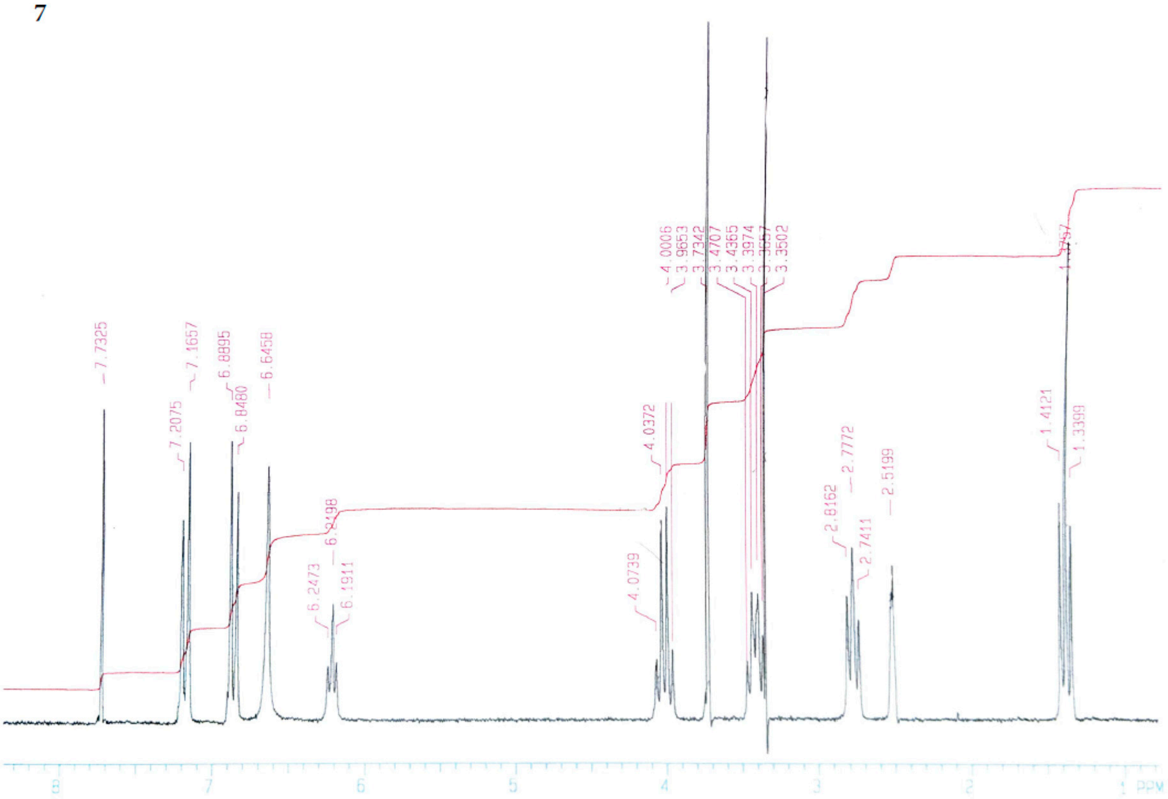

7

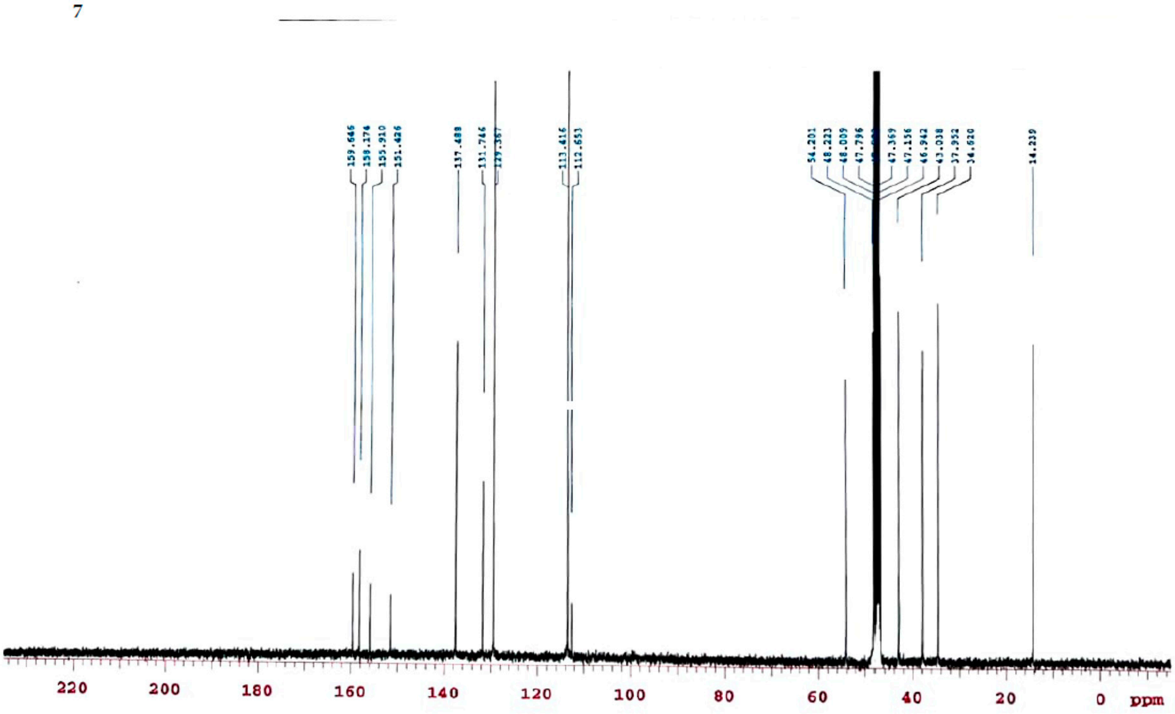

8

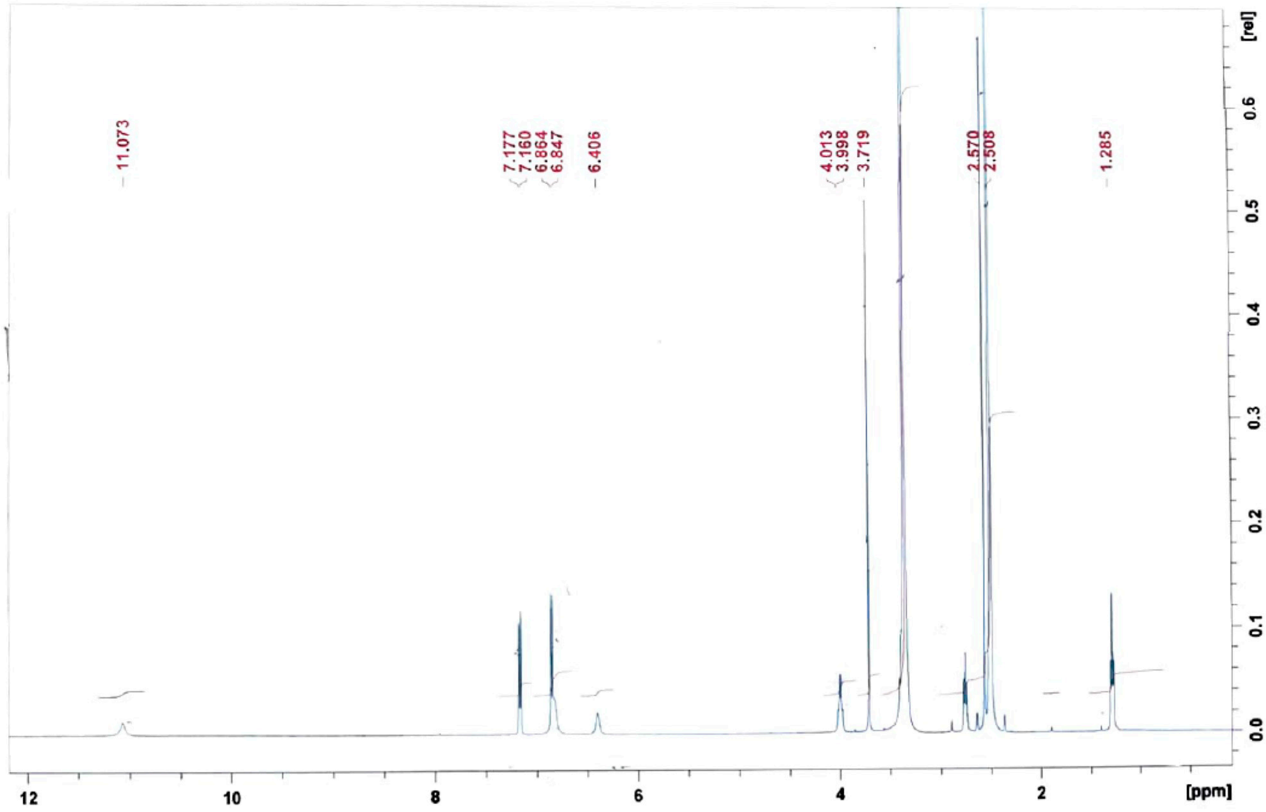
11.073
7.177
7.160
6.864
6.847
6.406
4.013
3.998
3.719
2.570
2.508
1.285
[rel]
[ppm]

8

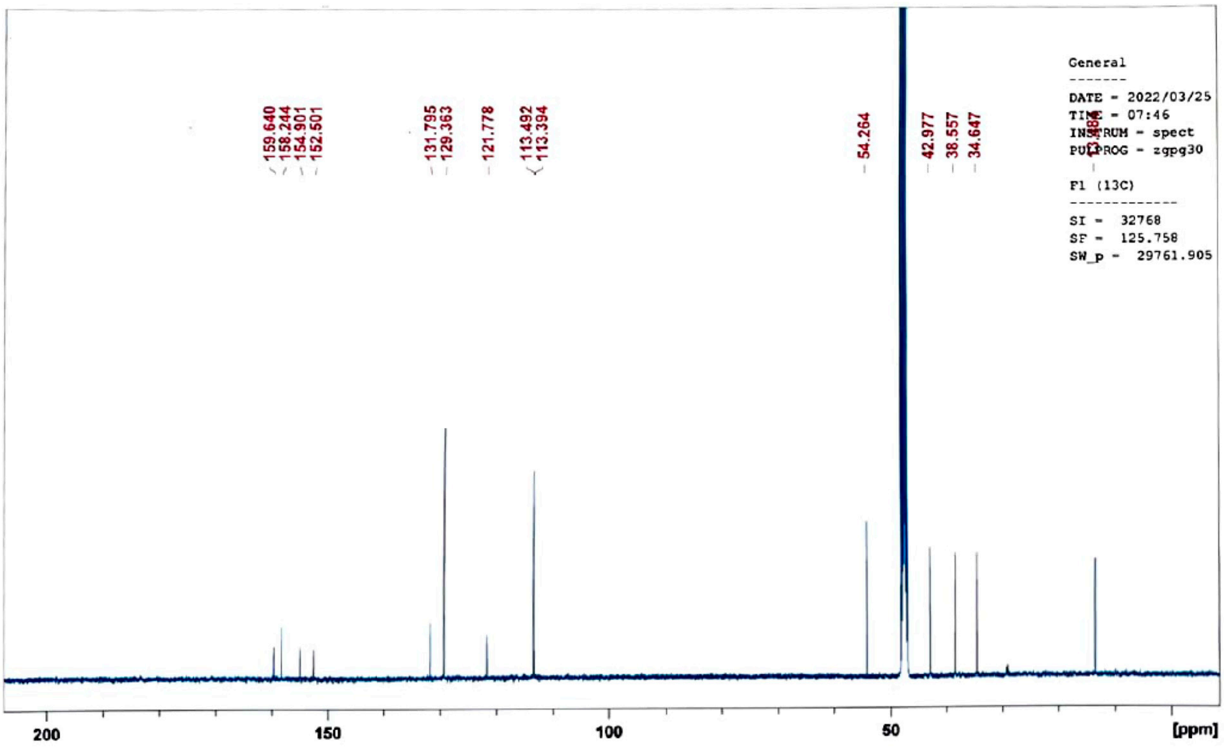
159.640
158.244
154.901
152.501
131.795
129.363
121.778
113.492
113.394
54.264
42.977
38.557
34.647
General
DATE - 2022/03/25
TI = 07:46
INSTRUM - spect
PU PROG - zgpg30
F1 (13C)
SI = 32768
SF = 125.758
SW_p = 29761.905
[ppm]

9

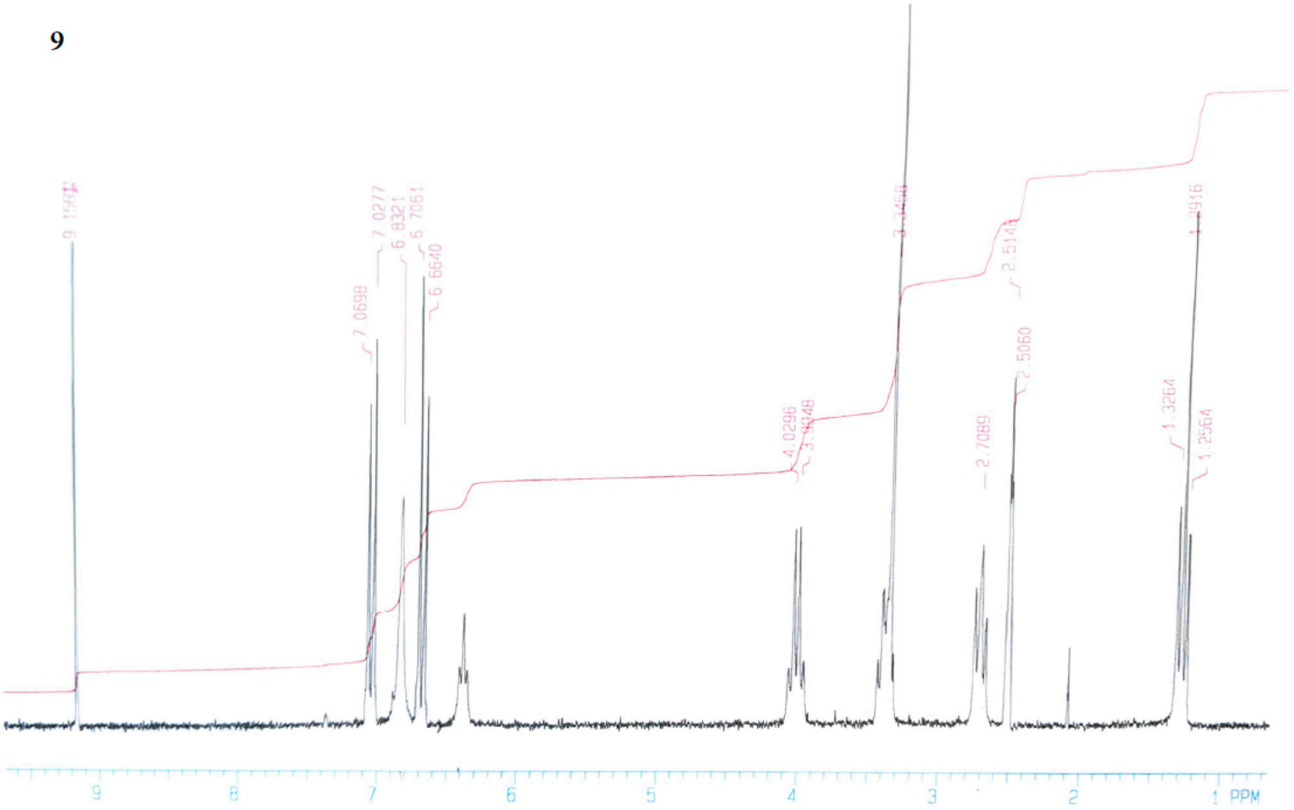
7.0698
7.0277
6.8321
6.7061
6.6640
4.0296
2.7089
2.5060
1.3264
1.2916
1.2564
9
8
7
6
5
4
3
2
1 PPM

9

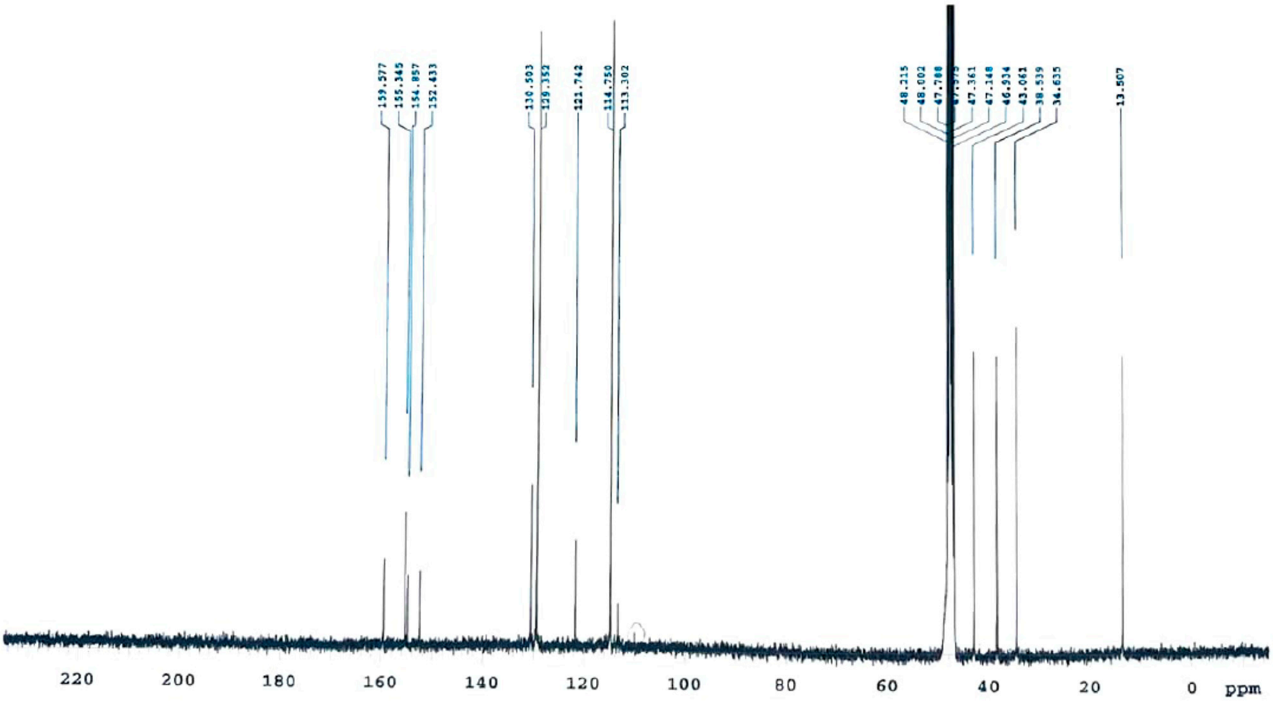
159.577
155.345
154.857
152.433
130.503
129.352
121.742
114.750
113.302
48.215
48.002
47.788
47.361
47.148
46.934
43.061
38.539
34.635
13.507
220
200
180
160
140
120
100
80
60
40
20
0
ppm

10

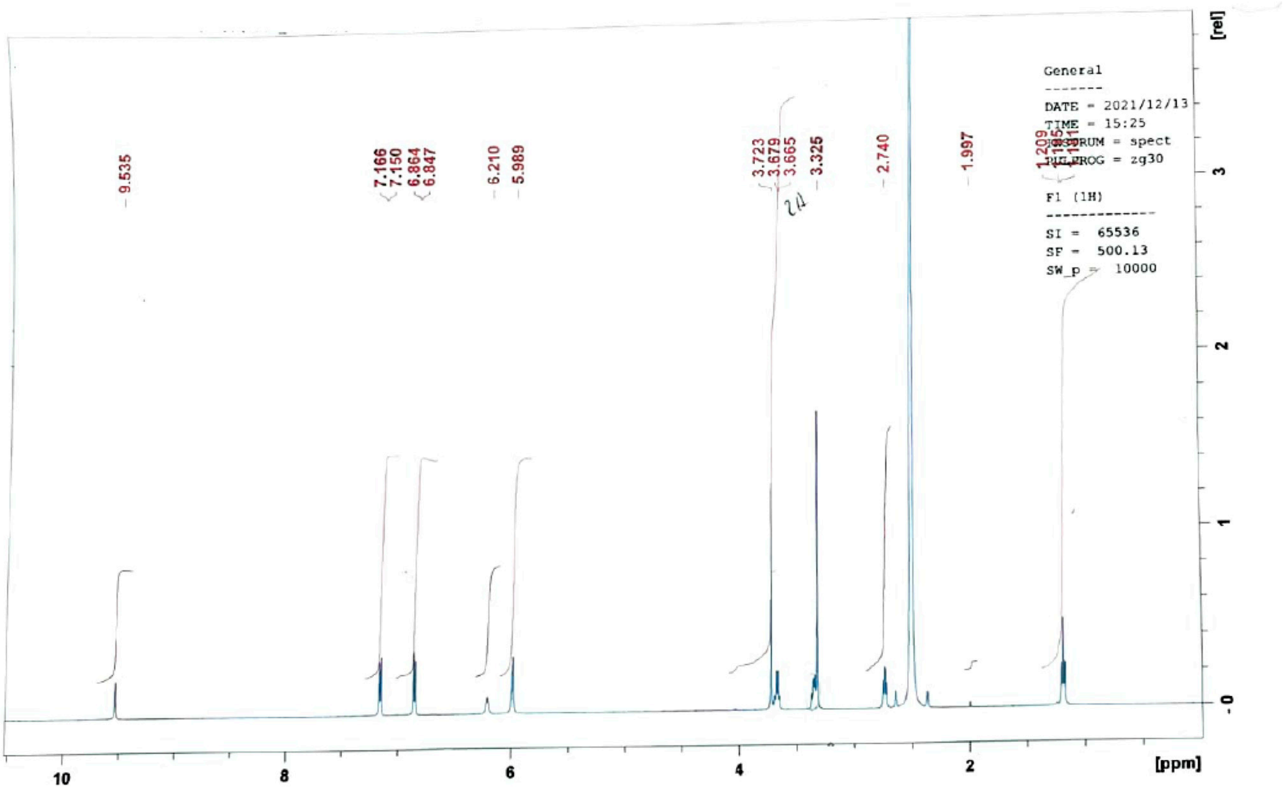
9.535
7.166
7.150
6.864
6.847
6.210
5.989
3.723
3.679
3.665
3.325
2.740
1.997
General
DATE = 2021/12/13
TIME = 15:25
PULPROG = zg30
F1 (1H)
SI = 65536
SF = 500.13
SW_p = 10000
[rel]
[ppm]
10
8
6
4
2

10

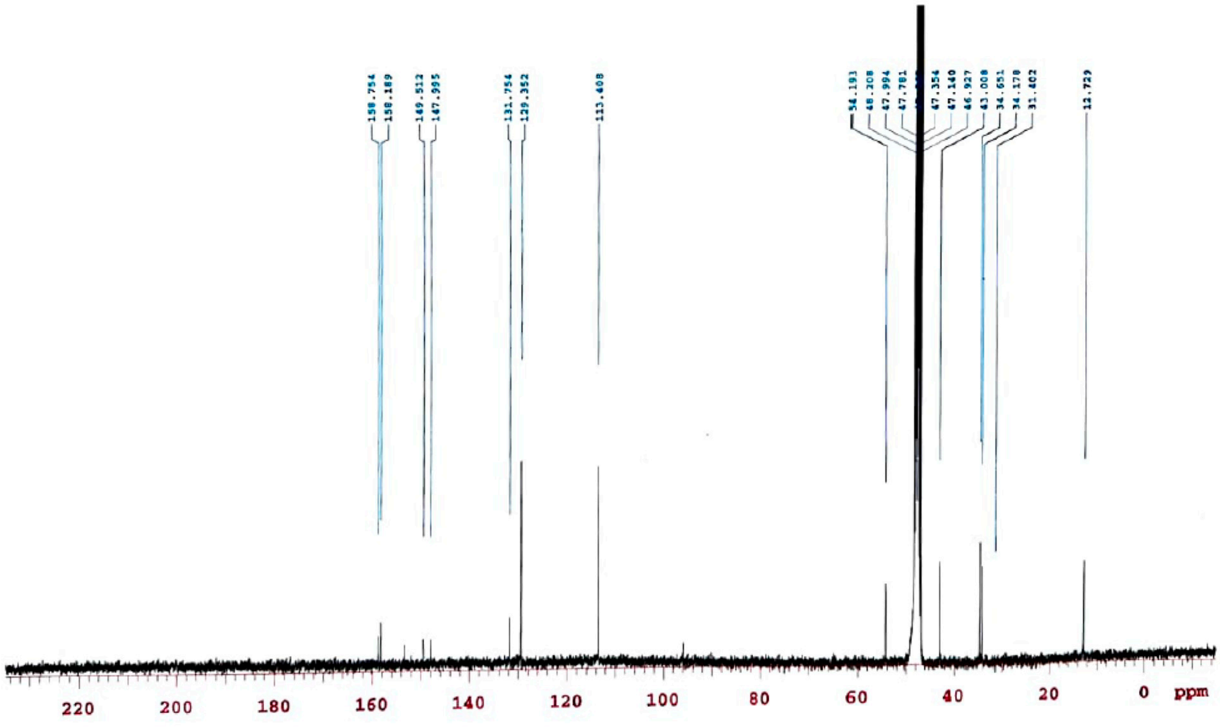
158.754
158.189
149.512
147.995
131.754
129.352
113.408
54.193
48.208
47.994
47.781
47.354
47.140
46.927
43.008
34.651
34.178
31.402
12.729
220
200
180
160
140
120
100
80
60
40
20
0
ppm

11

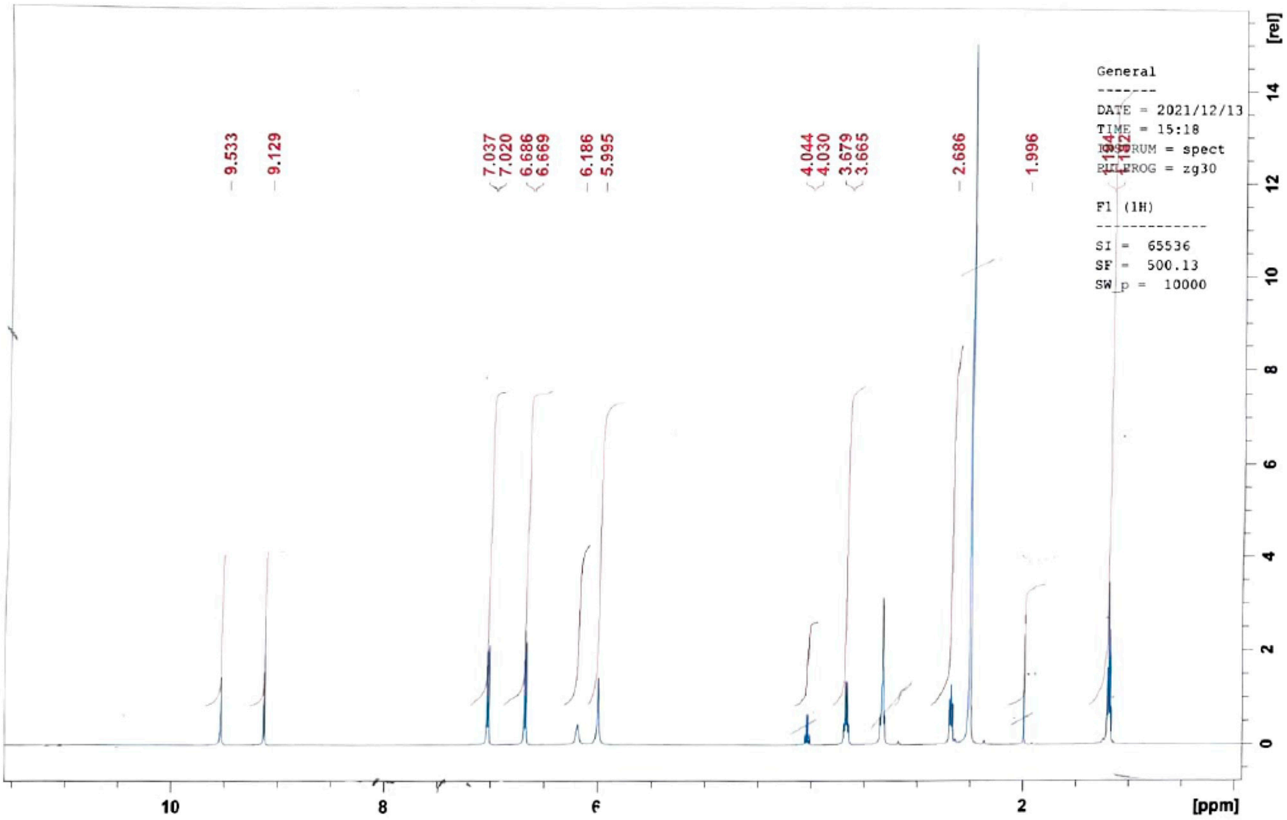
9.533
9.129
7.037
7.020
6.686
6.669
6.186
5.995
4.044
4.030
3.679
3.665
2.686
1.996
General
DATE = 2021/12/13
TIME = 15:18
PULPROG = zg30
F1 (1H)
SI = 65536
SF = 500.13
SW_p = 10000
[rel]
[ppm]

11

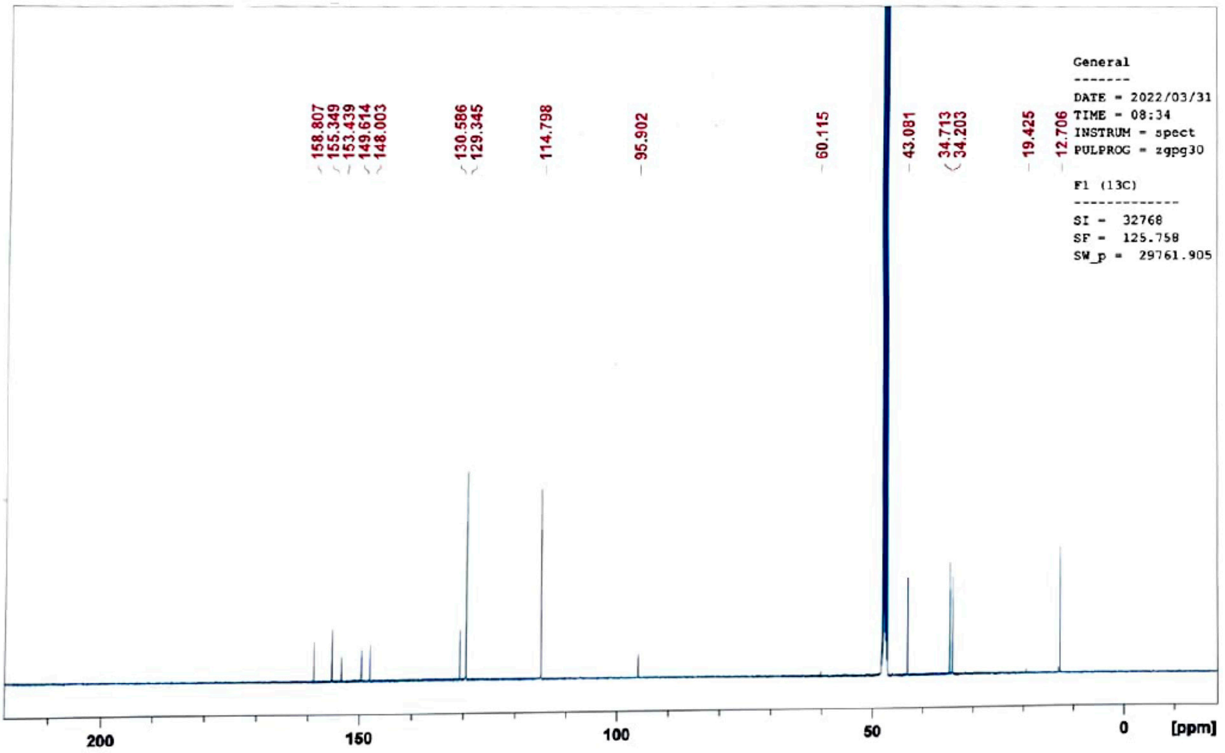
158.807
155.349
153.439
149.614
148.003
130.586
129.345
114.798
95.902
60.115
43.081
34.713
34.203
19.425
12.706
General
DATE = 2022/03/31
TIME = 08:34
INSTRUM = spect
PULPROG = zgpg30
F1 (13C)
SI = 32768
SF = 125.758
SW_p = 29761.905
[ppm]

12

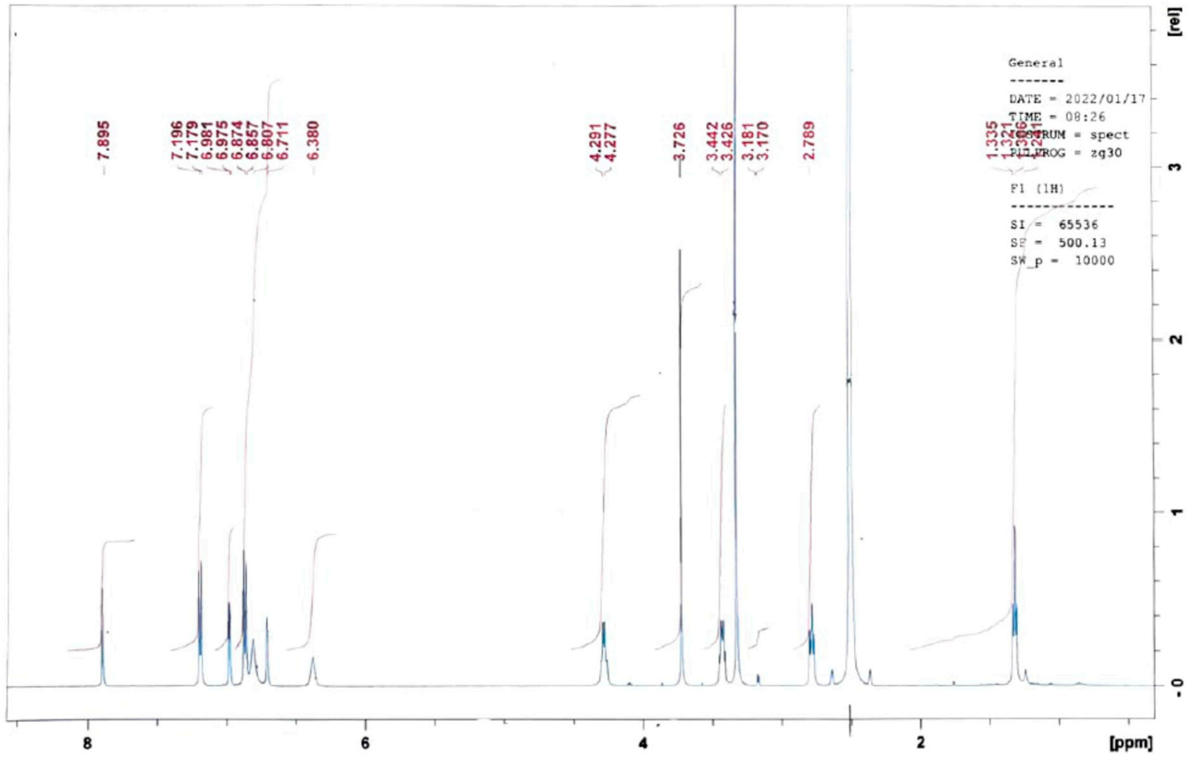
7.895
7.196
7.179
6.981
6.975
6.874
6.857
6.807
6.711
6.380
4.291
4.277
3.726
3.442
3.426
3.181
3.170
2.789
1.335
General
DATE = 2022/01/17
TIME = 08:26
PULPROG = zg30
F1 (1H)
SI = 65536
SF = 500.13
SW_p = 10000
[rel]
[ppm]

12

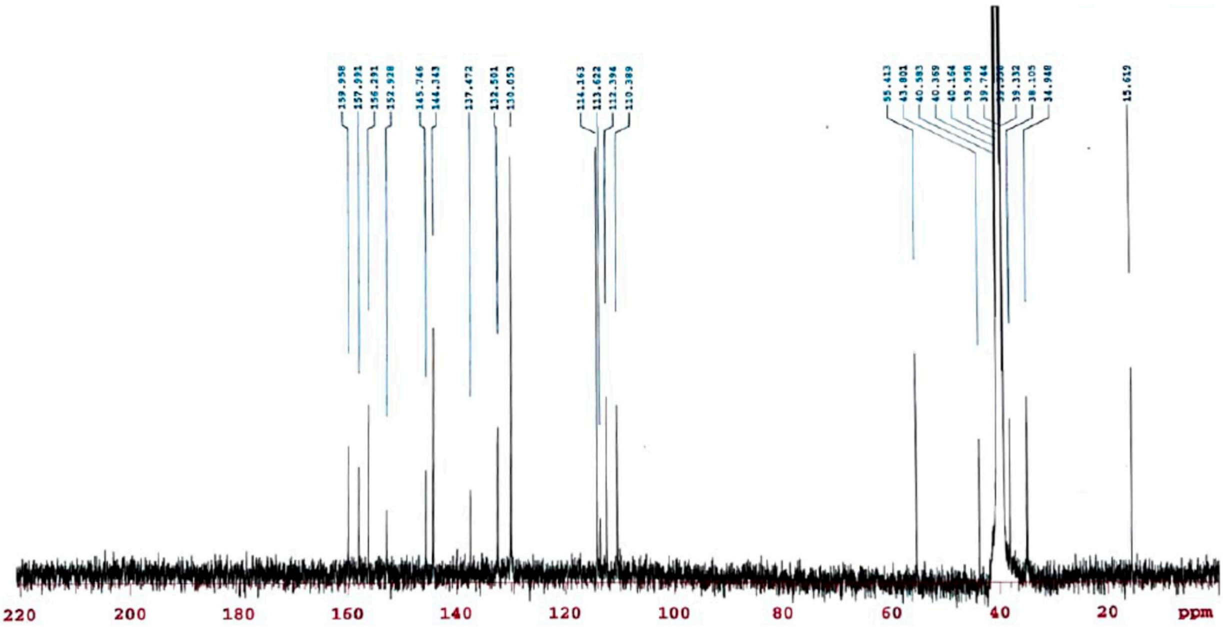
159.958
157.991
156.291
152.928
145.746
144.343
137.472
132.501
130.053
114.163
113.622
112.394
110.389
55.413
43.801
15.619
ppm

13

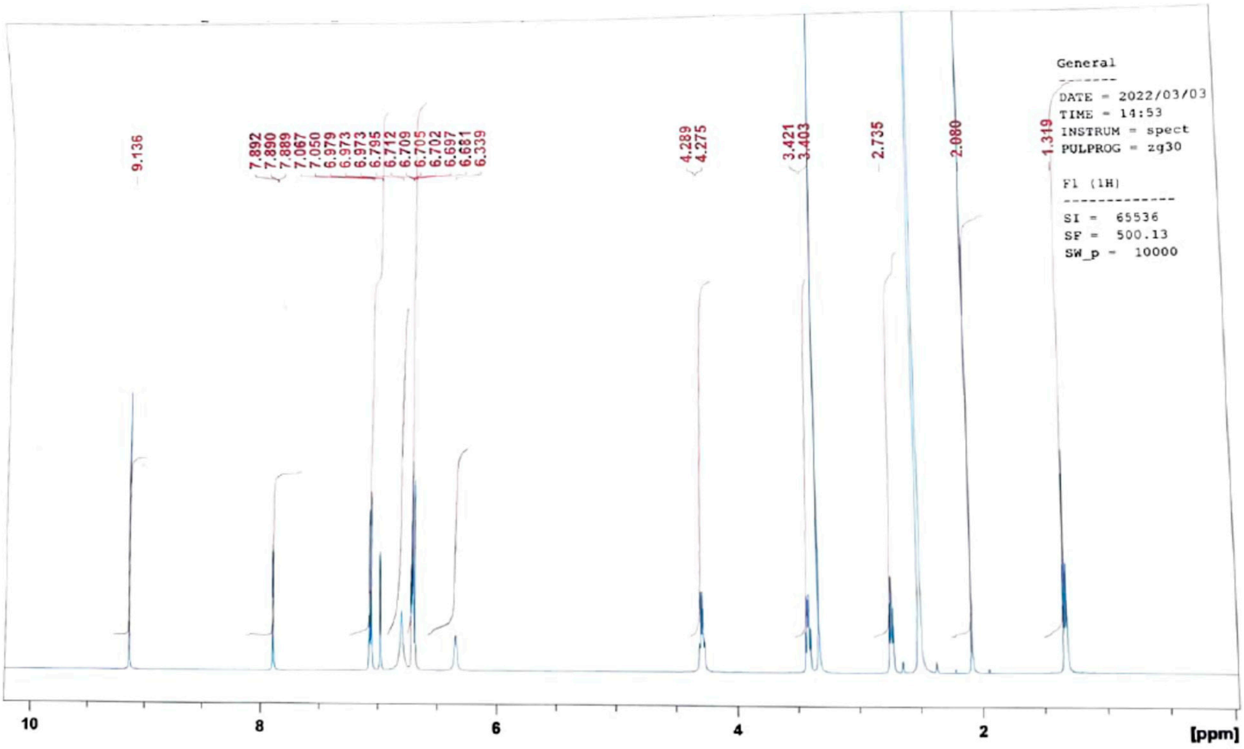

9.136
7.892
7.890
7.889
7.067
7.050
6.979
6.973
6.973
6.973
6.795
6.712
6.709
6.705
6.702
6.697
6.681
6.339
4.289
4.275
3.421
3.403
2.735
2.080
1.319
General
DATE = 2022/03/03
TIME = 14:53
INSTRUM = spect
PULPROG = zg30
F1 (1H)
SI = 65536
SF = 500.13
SW_p = 10000
10
8
6
4
2
[ppm]

13

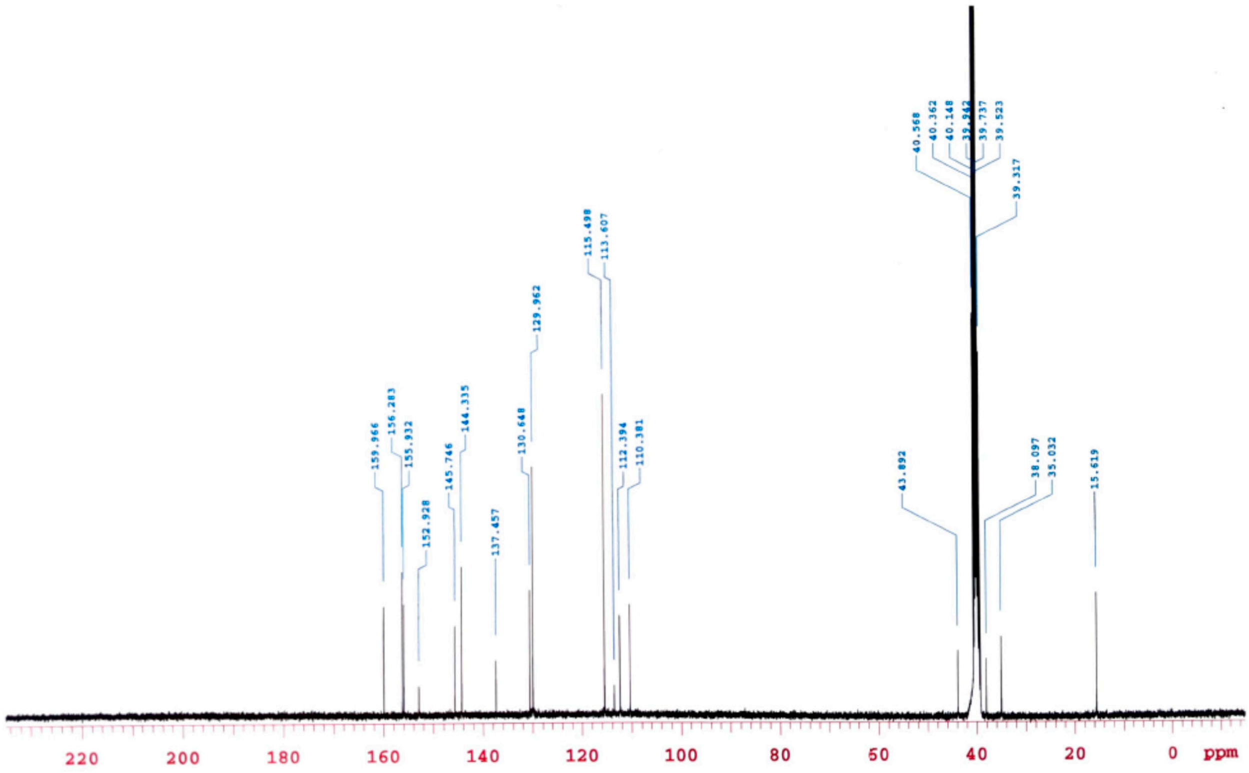

159.966
156.283
155.932
152.928
145.746
144.335
137.457
130.648
129.962
115.498
113.607
112.394
110.381
43.892
40.568
40.362
40.148
39.942
39.737
39.523
39.317
38.097
35.032
15.619
220
200
180
160
140
120
100
80
50
40
20
0
ppm

17

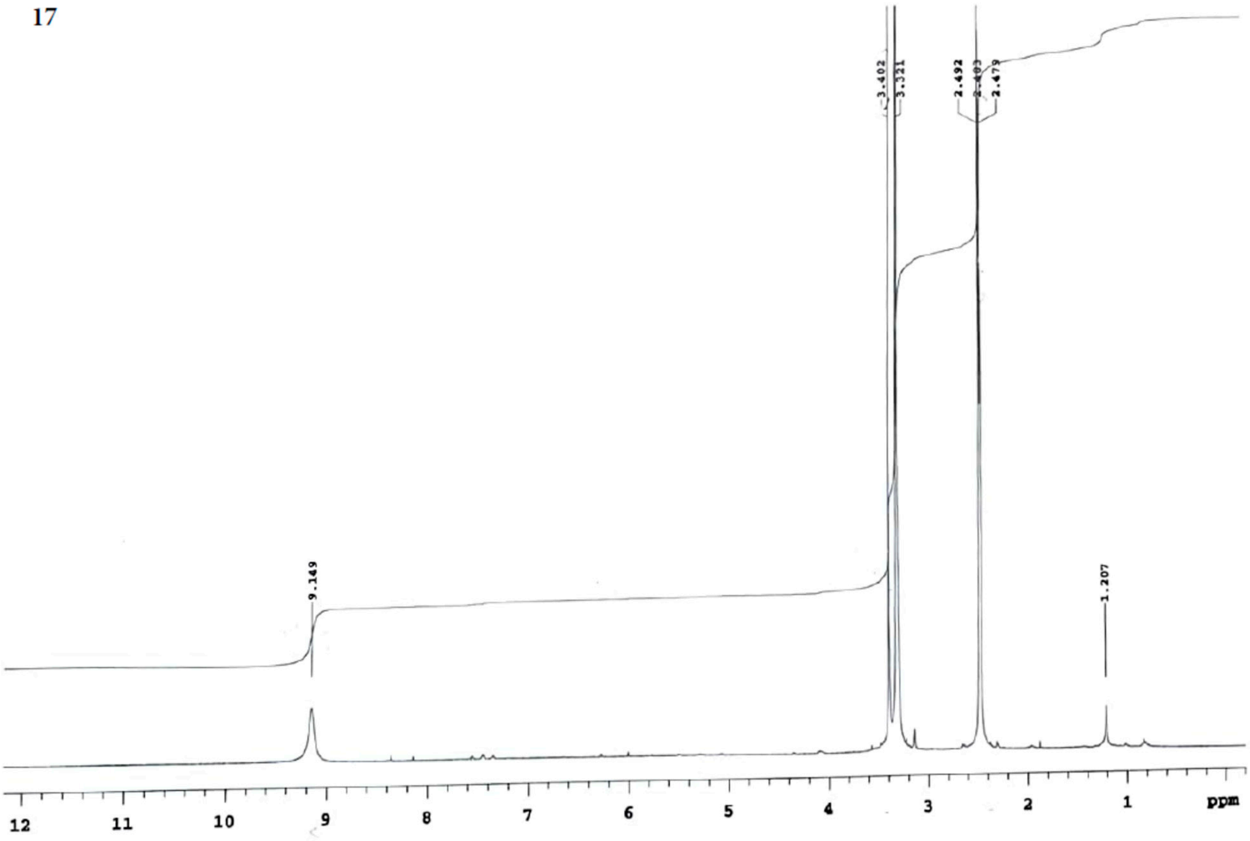

17

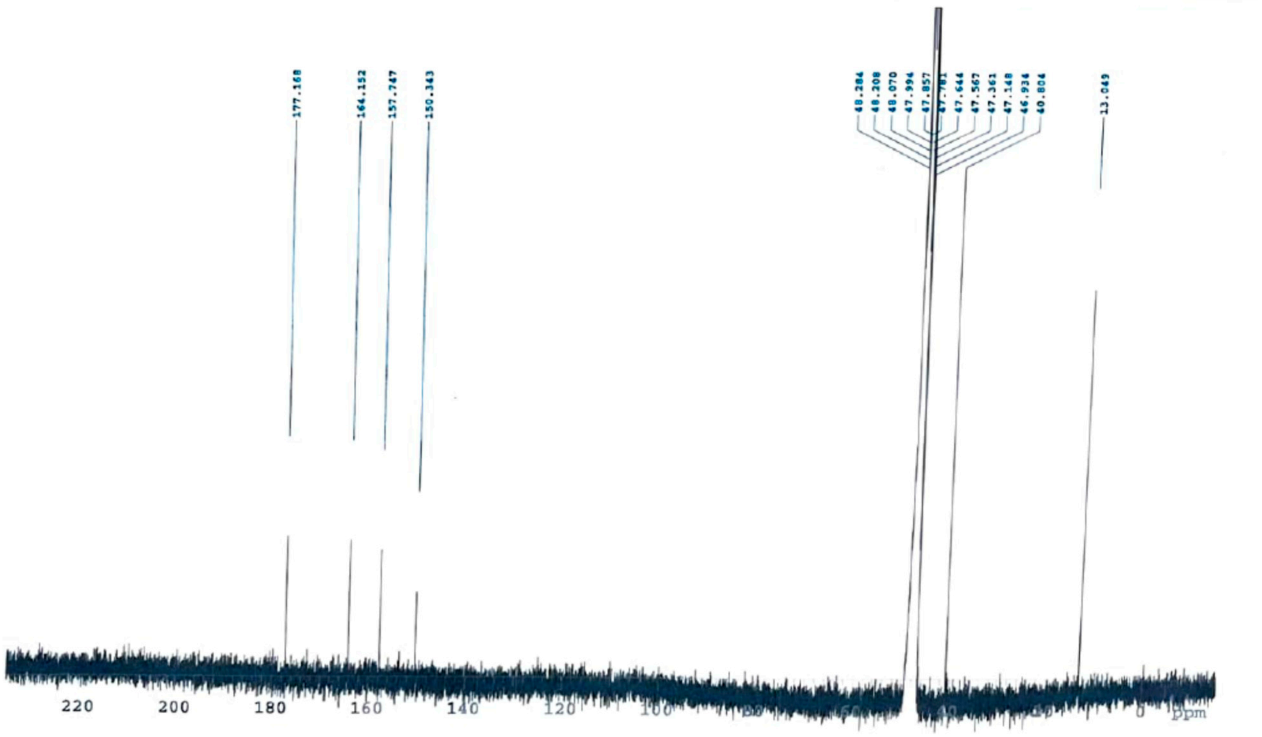

18

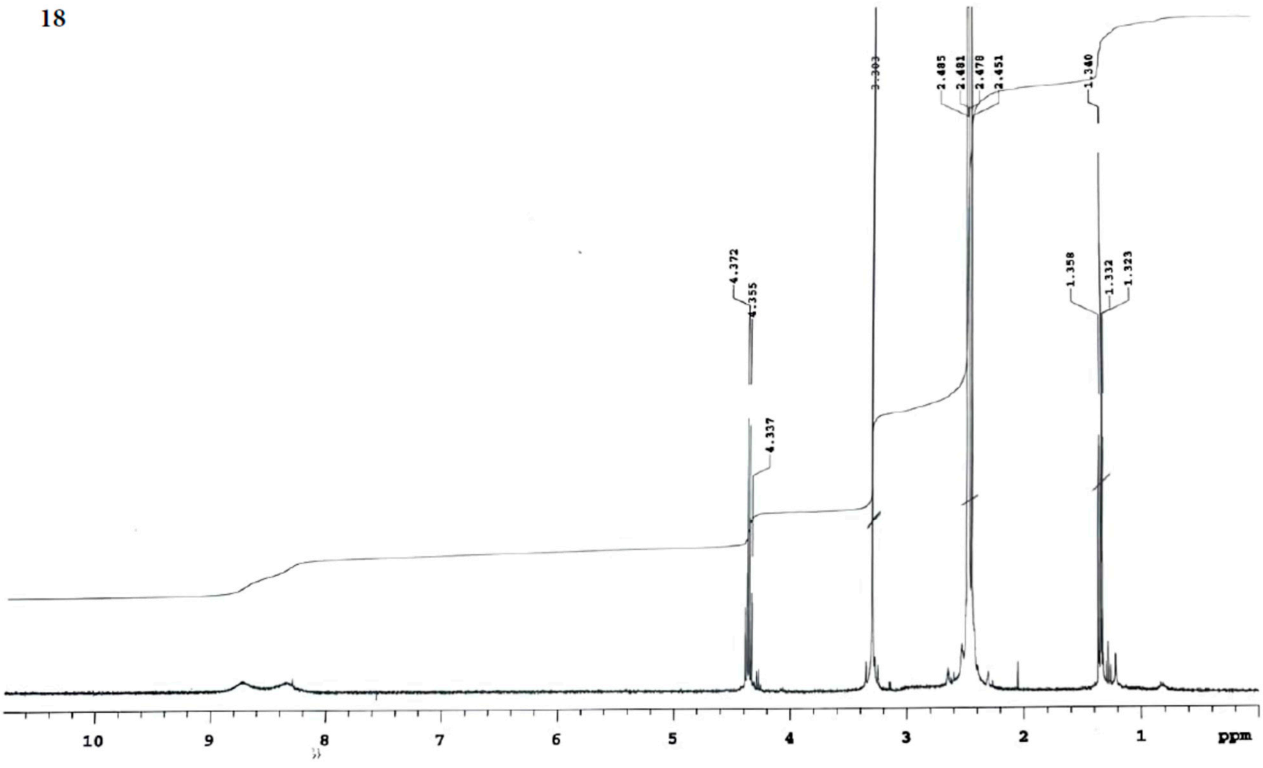
4.372
4.355
4.337
3.303
2.485
2.481
2.478
2.451
1.340
1.358
1.332
1.323
10
9
8
7
6
5
4
3
2
1
ppm

18

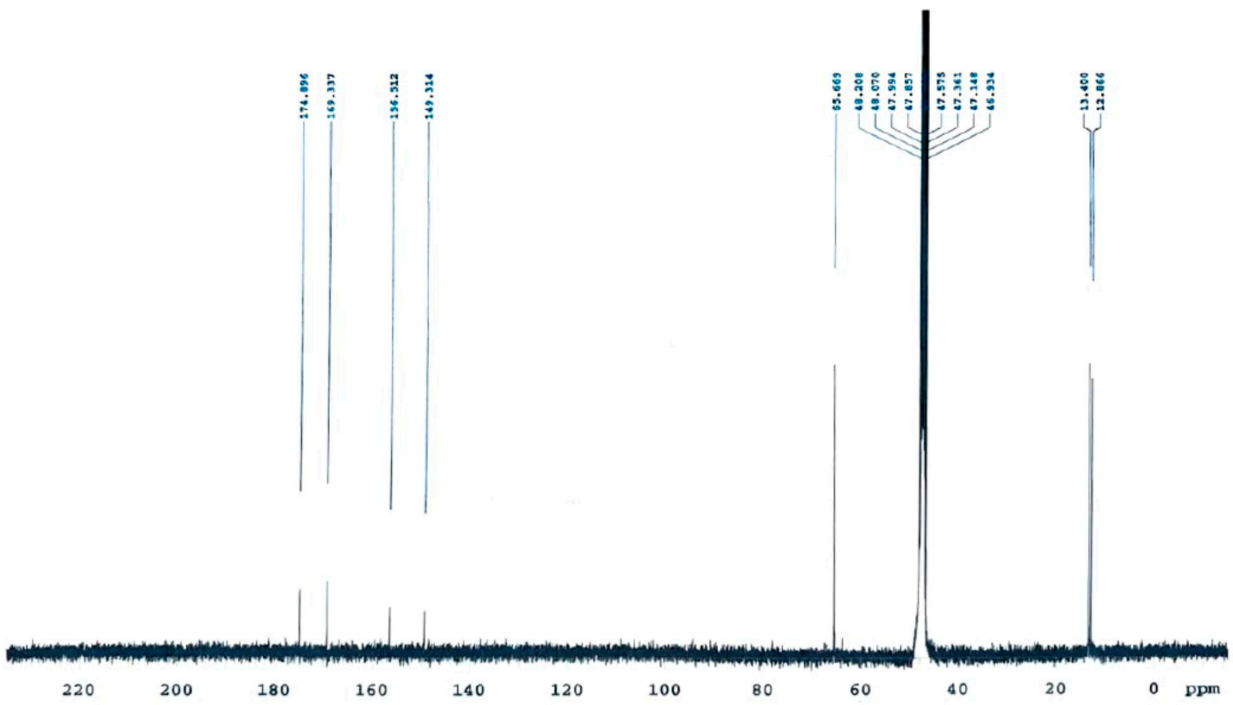
174.896
169.337
156.512
149.314
65.669
48.208
48.070
47.994
47.857
47.575
47.361
47.148
46.934
13.400
12.866
220
200
180
160
140
120
100
80
60
40
20
0
ppm

19

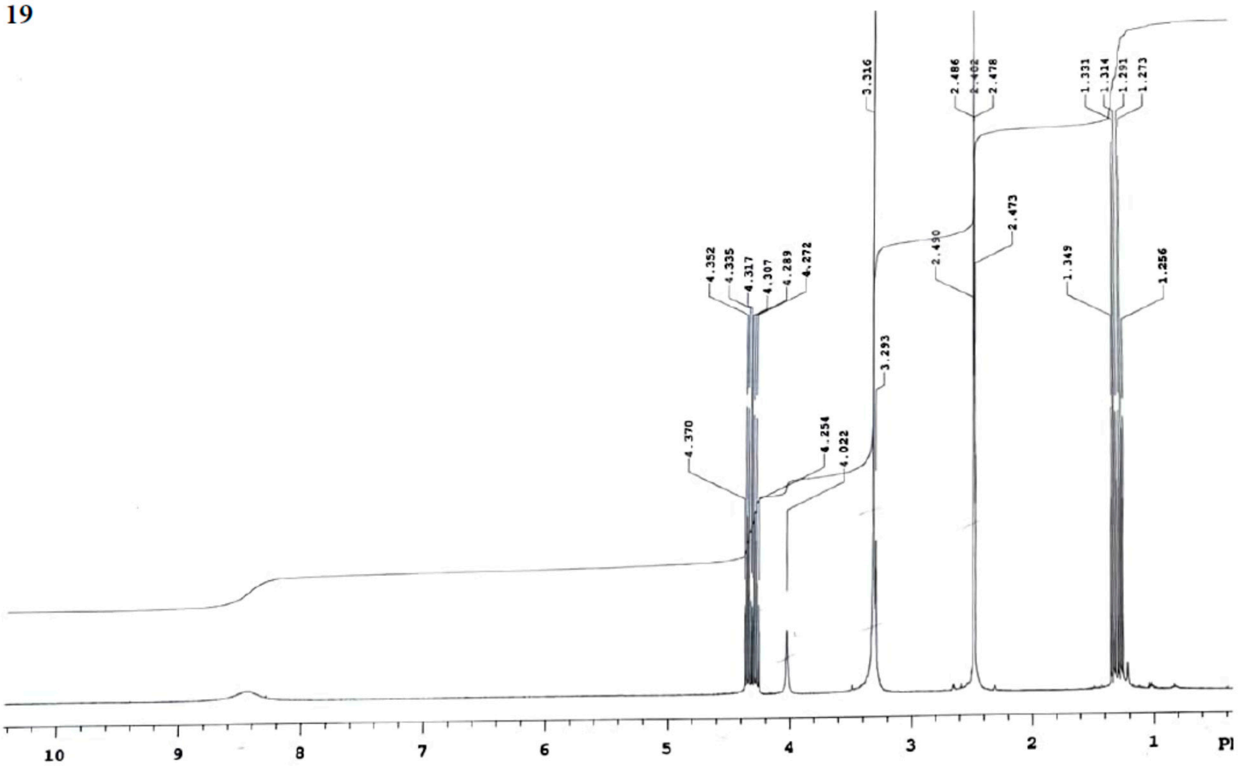
3.316
2.486
2.478
1.331
1.314
1.291
1.273
4.352
4.335
4.317
4.307
4.289
4.272
2.490
2.473
1.349
1.256
3.293
4.370
4.254
4.022
10
9
8
7
6
5
4
3
2
1
Pl

20

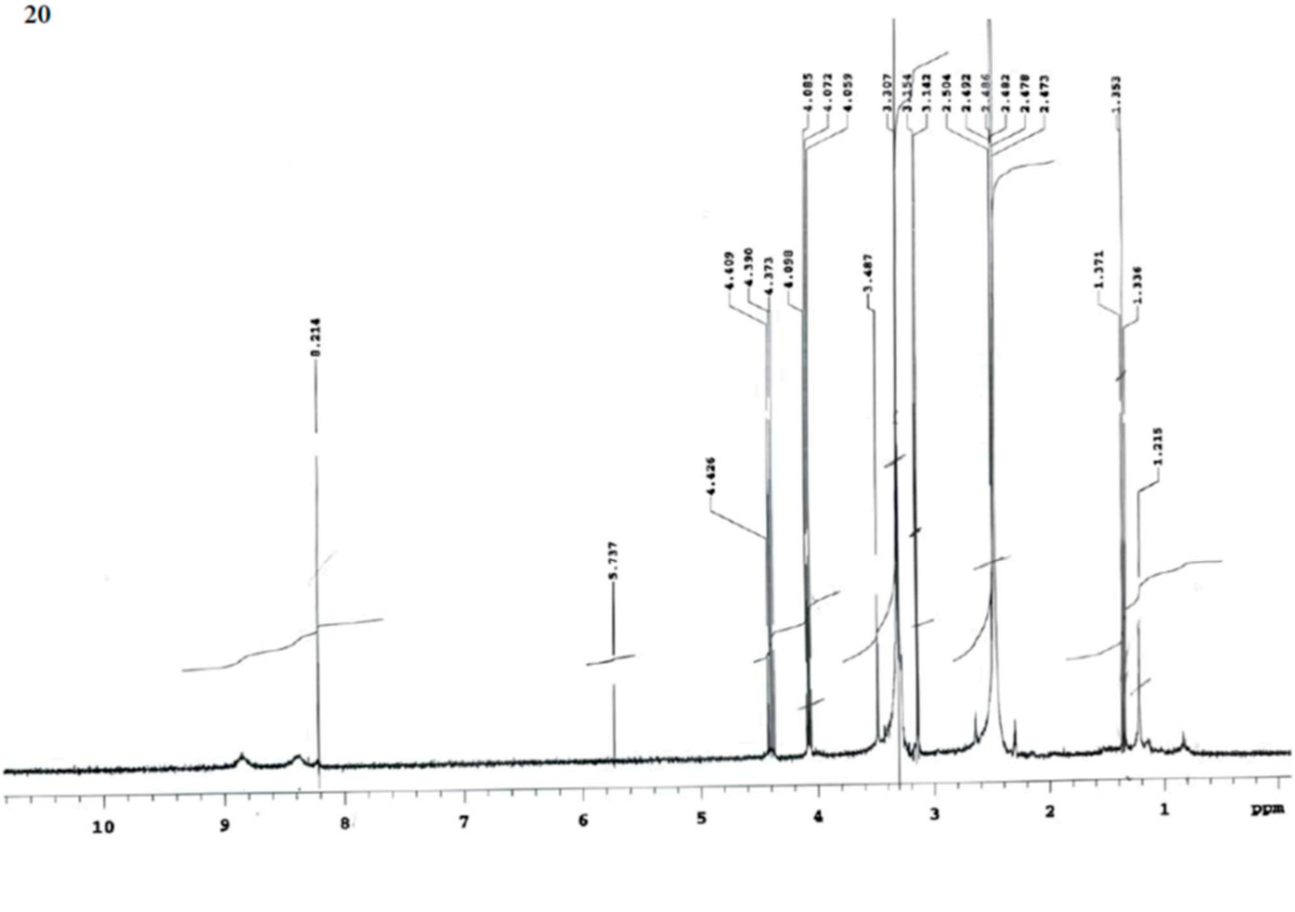
4.085
4.072
4.059
3.307
3.154
3.142
2.504
2.492
2.486
2.482
2.478
2.473
1.353
4.409
4.390
4.373
4.098
3.487
1.371
1.336
8.214
1.215
4.426
5.737
10
9
8
7
6
5
4
3
2
1
ppm

20

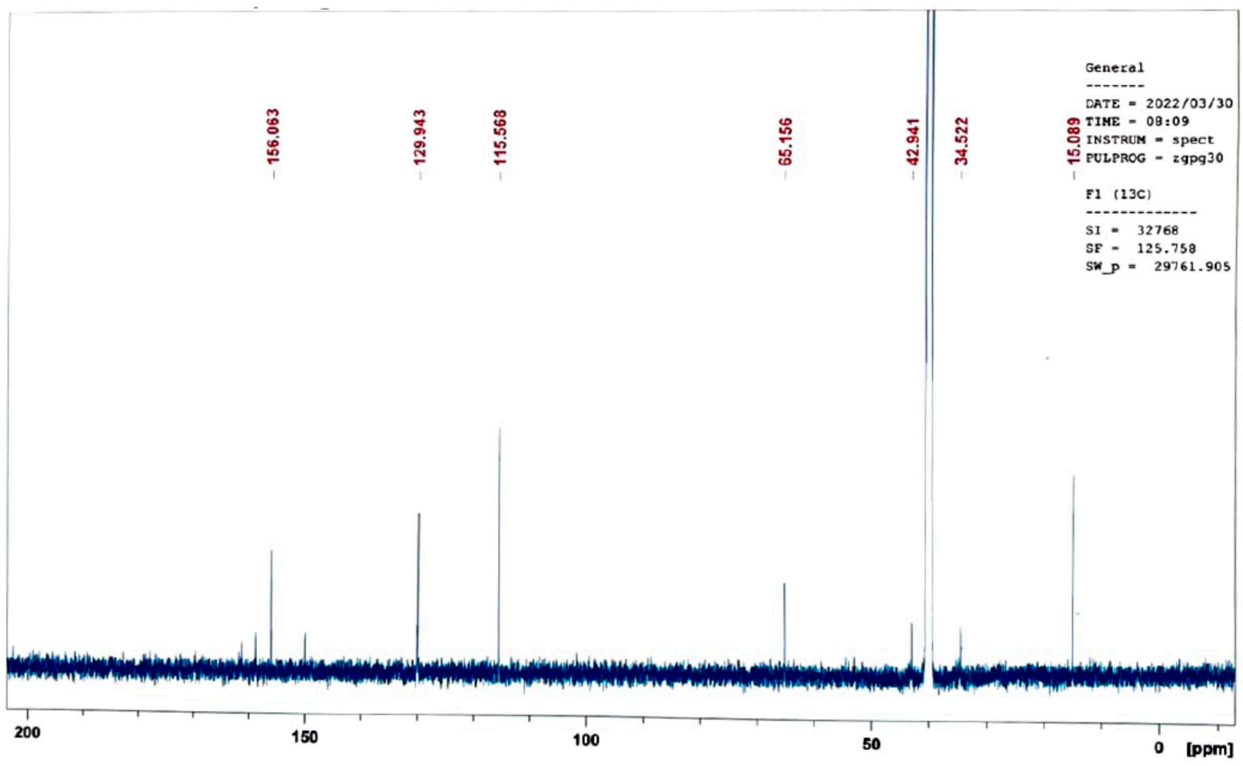
156.063
129.943
115.568
65.156
42.941
34.522
15.089
General

DATE = 2022/03/30
TIME = 08:09
INSTRUM = spect
PULPROG = zgpg30
F1 (13C)

SI = 32768
SF = 125.758
SW_p = 29761.905
200
150
100
50
0
[ppm]

21

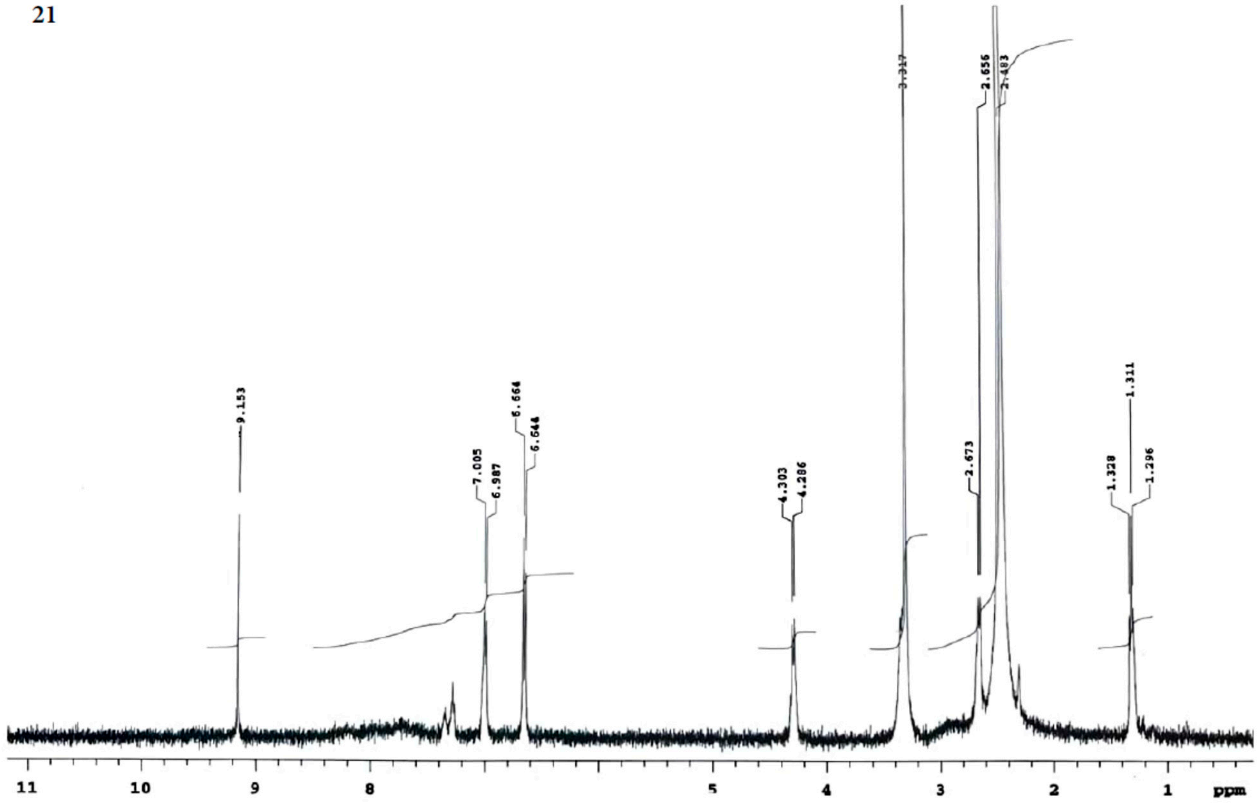
9.153
7.005
6.987
6.664
6.544
4.303
4.286
3.317
2.673
2.656
2.483
1.328
1.311
1.296
11
10
9
8
5
4
3
2
1
ppm

21

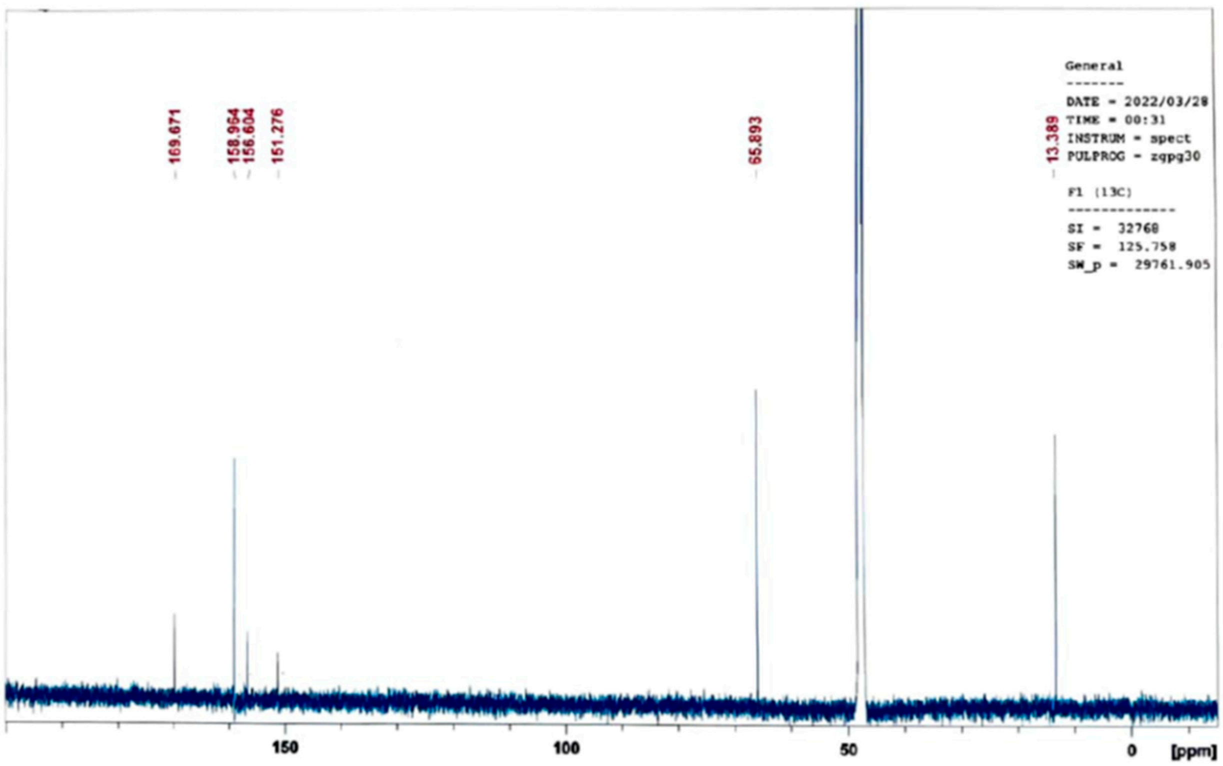
169.671
158.964
156.604
151.276
65.893
13.389
General
DATE = 2022/03/28
TIME = 00:31
INSTRUM = spect
PULPROG = zgpg30
F1 (13C)
SI = 32768
SF = 125.758
SW_p = 29761.905
150
100
50
0
[ppm]
